# High-throughput engineering of bispecific antibodies to enhance macrophage-mediated cytotoxicity of B-cell lymphoma

**DOI:** 10.1038/s41467-026-76180-5

**Published:** 2026-08-01

**Authors:** Carlota Pagès-Geli, Juliano Ribeiro, Thomas Wienclaw, Anna M. Meglan, Lauren Sloat, Matheus Silva, Jasmine Blandin, José Velarde, Kyle Vaccaro, Carolin Sebastiany, Patricia Fernández-Guzmán, Cynthia K. Hahn, Marta Crespo, Kipp Weiskopf

**Affiliations:** 1 Department of Medicine, Beth Israel Deaconess Medical Center, Boston, MA USA; 2 Department of Medicine, Harvard Medical School, Boston, MA USA; 3 Harvard-MIT Health Sciences and Technology Program, Boston, MA USA; 4 Whitehead Institute for Biomedical Research, Cambridge, MA USA; 5 Experimental Hematology, Vall d’Hebron Institute of Oncology (VHIO), Vall d’Hebron Barcelona Hospital Campus, C/ Natzaret, 115-117, Barcelona, Spain; 6 Department of Medicine, Universitat Autònoma de Barcelona, Bellaterra, Spain; 7 Department of Medical Oncology, Dana-Farber Cancer Institute, Boston, MA USA; 8 The Broad Institute of MIT and Harvard, Cambridge, MA USA; 9 Cancer Center, Beth Israel Deaconess Medical Center, Boston, MA USA; 10 Cancer Research Institute, Beth Israel Deaconess Medical Center, Boston, MA USA

**Keywords:** Target identification, Drug development, Lymphoma, Monocytes and macrophages, Antibody therapy

## Abstract

Macrophages are critical effectors of antibody therapies for lymphoma, but the best targets to engage their function remain unknown. Here, we develop a ‘tactical surfaceome profiling’ strategy to define a comprehensive repertoire of surface antigens on B-cell lymphoma that can be targeted with antibodies to provoke macrophage attack. Across both mouse and human systems, we identify multiple unappreciated targets of opsonization as well as putative immune checkpoints. We incorporate this information into a high-throughput engineering strategy, creating 156 bispecific antibodies and identifying dozens that stimulate macrophage-mediated cytotoxicity. A heterodimeric scFv-Fc format enables our approach; it is superior to conventional bispecific antibody design and offers enhanced activity while reducing developmental complexity. Among the therapeutics we create, a bispecific comprising a SIRPα decoy domain and a CD38-targeting arm (WTa2d1xCD38) exhibits maximal efficacy with reduced risk of toxicity. This bispecific stimulates robust anti-tumor responses in xenograft models of aggressive B-cell lymphoma and shows benefits over anti-CD20 antibody rituximab. Our approach can be applied to other cancers to leverage anti-tumor responses by macrophages or other immune cells.

## Abstract (web summary)

B-cell lymphomas can resist macrophage-mediated phagocytosis through different mechanisms. Here the authors present the results of a high-throughput system to screen for antibodies enhancing macrophage-mediated cytotoxicity of B-lymphoma cells, proposing a bispecific antibody comprising a SIRPα decoy domain and a CD38 targeting arm as therapeutic candidate.

## Introduction

B-cell non-Hodgkin lymphoma (NHL) is the most common hematologic malignancy worldwide and remains a large, unmet clinical need. It is a highly heterogeneous disease, with multiple subtypes distinguished by variations in both genetic alterations and the immune microenvironment^1,2^. Diffuse large B-cell lymphoma (DLBCL) is the most common subtype and accounts for approximately 35% of all B-cell lymphomas. Despite advances in frontline rituximab-based chemoimmunotherapy (e.g., R-CHOP), a significant proportion of patients become refractory or relapse after the first line of treatment^3^. Subsequent treatment strategies have focused on promoting T-cell cytotoxicity, such as CAR T-cell therapy and bispecific T-cell engagers. However, these therapies only cure a subset of patients^4,5^, and many patients exhibit inadequate T-cell infiltration and function^6–8^. Additionally, more than 50% of B-cell lymphomas exhibit genetic alterations that allow them to evade recognition by T cells, such as downregulation or loss of the Major Histocompatibility Complex (MHC) antigen presentation machinery or overexpression of immune checkpoint receptors^9^. These features may limit opportunities for T-cell-directed therapies while creating vulnerabilities for orthogonal approaches that target innate immune cells, particularly macrophages^10^.

In B-cell lymphoma, the presence of CD68+ macrophages was linked to a favorable prognosis in patients treated with R-CHOP, while it correlated with poor outcomes in the absence of rituximab^11^. Antibody-dependent cellular phagocytosis (ADCP) is a major mechanism that contributes to the efficacy of rituximab in vivo^12^, likely underlying this effect. However, ADCP is often limited by the CD47/SIRPα macrophage immune checkpoint. CD47 is a cell-surface antigen expressed by many normal and neoplastic tissues and acts as a “don’t eat me” signal to prevent macrophage phagocytosis^13^. Therapies that block the CD47/SIRPα interaction can enhance ADCP and synergize with rituximab in B-cell lymphoma preclinical models^14–16^. This therapeutic approach is under investigation in clinical trials for patients with B-cell malignancies^17,18^. Similarly, downregulation of MHC-I may make lymphoma cells more vulnerable to macrophage phagocytosis in the presence of tumor-opsonizing antibodies or CD47-blocking therapies^10^. Despite the success of monoclonal antibodies for hematologic malignancies, there have been limited efforts to identify additional antibodies that stimulate macrophages to attack lymphoma cells.

In this work, we develop a strategy to perform ‘tactical surfaceome profiling’ by screening comprehensive libraries of monoclonal antibodies against a diversity of cell-surface antigens, pairing each antibody's ability to bind lymphoma cells with its ability to elicit macrophage-mediated cytotoxicity. Our approach is target-agnostic and “function first” to identify and prioritize antibodies that robustly stimulate macrophages to eliminate lymphoma cells. We successfully perform screens across both the murine and human systems, then harmonize the hits for further validation. To perform a comprehensive evaluation of antibody combinations, we develop a high-throughput system to rapidly engineer and evaluate a matrix of bispecific antibodies (bsAbs). Overall, we identify therapeutic strategies and therapeutic agents to provoke macrophages to attack B-cell lymphoma. These efforts can be translated to the clinic, and we expect this high-throughput approach to be rapidly applied to identify new therapies for other types of cancer.

## Results

### Development of a high-throughput system to screen for macrophage-mediated cytotoxicity of B-cell lymphoma

**Fig. 1:**
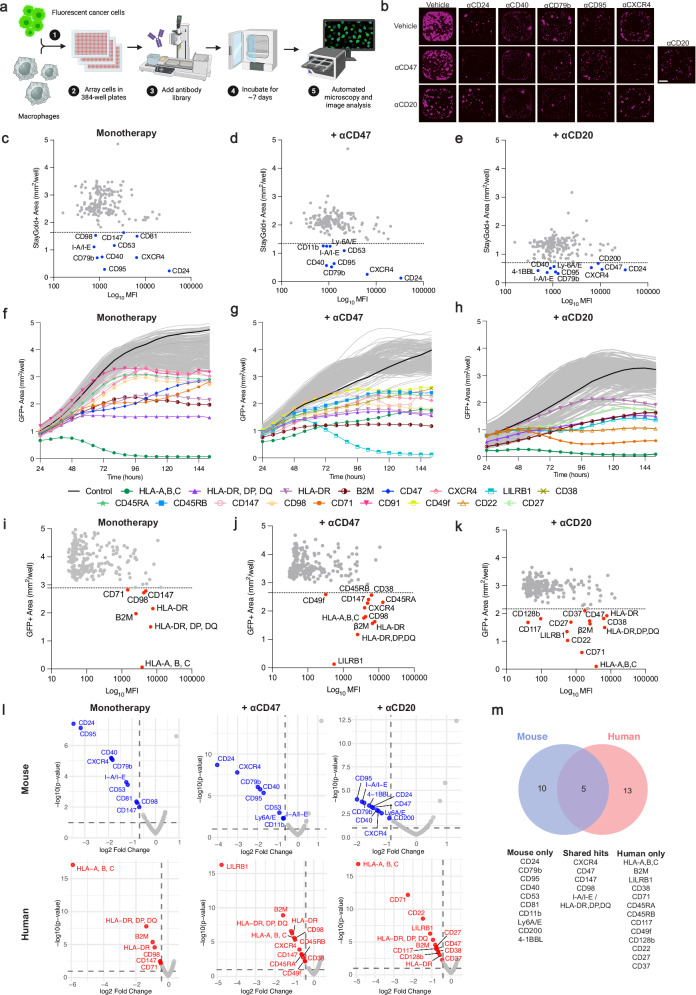
‘Tactical surfaceome profiling’ identifies targets for macrophage-directed immunotherapy of B-cell lymphoma. **a** Strategy to identify antibodies that provoke macrophage-mediated cytotoxicity of lymphoma. Murine or human macrophages were co-cultured with StayGold+ MHC-I KO A20 cells (murine B-cell lymphoma line) or GFP+ Raji cells (human Burkitt lymphoma line), respectively. Co-cultures were subjected to control (“monotherapy”), anti-CD47, or anti-CD20 treatment. Arrayed libraries of monoclonal antibodies targeting murine (*n* = 173) and human (*n* = 241) antigens were overlaid and the fluorescent area was quantified over time. Adapted from Vaccaro et al., *J Clin Invest* 2024^38^. Created in BioRender. Pagès-Geli, C. (2026) https://BioRender.com/jc918q3. **b** Whole-well images showing StayGold+ A20 lymphoma area (purple) from co-cultures treated with the indicated antibodies. Representative of 2 individual co-cultures per condition. Scale bar, 800 μm. **c–e** Comparison of each library antibody binding to A20 cells (x-axis) versus anti-lymphoma function at t = 152 h (y-axis). Lower fluorescent area indicates greater macrophage anti-lymphoma activity. Blue points highlight antibodies exceeding the 95th percentile (dashed line) in functional activity in the monotherapy (**c**), anti-CD47 (**d**), and anti-CD20 (**e**) conditions. **f–h** Growth of GFP+ human Raji lymphoma in co-culture with human macrophages under monotherapy (**f**), anti-CD47 (**g**), anti-CD20 (**h**) conditions. Each curve represents the mean growth of lymphoma cells treated with a different antibody from *n* = 2 individual co-cultures. Black, control; colored curves, representative hits. **i-–k** Comparison of each library antibody binding to GFP+ Raji cells (x-axis) versus anti-lymphoma function at t = 152 h (y-axis). Lower fluorescent area indicates greater macrophage anti-lymphoma activity. Red points highlight antibodies exceeding the 95th percentile (dashed line) in functional activity in monotherapy (**i**), anti-CD47 (**j**), and anti-CD20 (**k**) conditions. (**l**) Volcano plots depicting hits based on absolute log2(FC) exceeding the 95th percentile and exploratory statistical analysis (*p* ≤ 0.05, one-tailed t-test, unadjusted) across the murine (blue) and human (red) systems. **m** Summary of hits identified in the murine and/or human systems. **c–l** Data represents mean of *n* = 2 individual co-cultures per condition. Binding data (MFI) performed once for mouse (**c**,**d**) and human (**i–k**) antigens. Source data are provided as a Source Data file.

To identify unappreciated targets for macrophage-directed immunotherapy of B-cell lymphoma, we developed a high-throughput assay to screen antibodies for their ability to stimulate macrophage-mediated cytotoxicity of lymphoma cells (Fig. 1a). We first differentiated mouse bone marrow cells into macrophages using M-CSF, and co-cultured them with fluorescent A20 MHC-I KO lymphoma cells. We employed an MHC-I KO line to model intrinsic resistance to T cell-directed killing^19–21^ and focus on opportunities for innate immune intervention. To the co-cultures, we added an arrayed library of 173 purified monoclonal antibodies (Supplementary Table 1) targeting different murine cell-surface antigens. We tested each antibody in duplicate across three different treatment conditions: (i) monotherapy, (ii) combination with anti-CD47, or (iii) combination with anti-CD20. We performed automated microscopy and image analysis over a 7-day period to quantify the fluorescent area as a metric of lymphoma cell growth or elimination. For each treatment condition, we identified the antibodies that stimulated the greatest anti-tumor function. Consistently across all conditions, we observed that antibodies to CD24, CD95 (FAS), CD40, CXCR4, and CD79b were able to elicit robust macrophage-mediated cytotoxicity of the A20 cells (Fig. 1b–e). We also performed quantitative surfaceome profiling by evaluating the ability of each antibody to bind to the surface of the A20 cells by flow cytometry. We observed a modest trend between antibody binding and the anti-tumor response by macrophages (Fig. 1c–e). For example, CD24 was the highest detected surface antigen in our assay, and it induced the greatest anti-tumor response. However, many targets existed as outliers, suggesting that the degree of binding to lymphoma cells alone is not sufficient to predict whether an antibody can elicit macrophage-mediated cytotoxicity. Furthermore, our results indicate that some antibodies may preferentially act on the macrophages in the co-culture system (e.g., Ly-6A/E, 4-1BBL), suggesting they modulate immune checkpoints (Supplementary Fig. 1). In total, these studies identified several unappreciated targets for innate immunotherapy of A20 cells that may present therapeutic opportunities for lymphoma.

### Discovery of targets and antibodies for macrophage-mediated cytotoxicity of human B-cell lymphoma

To understand whether we could similarly identify targets of antibody-dependent phagocytosis in the human system, we also evaluated an arrayed library of 241 purified monoclonal antibodies (Supplementary Table 1) targeting different human cell-surface antigens. The library included antibodies to 92 antigens that were shared with the murine library (Supplementary Fig. 2a). We differentiated primary human monocytes ex vivo to macrophages using M-CSF, and then we co-cultured them with GFP+ Raji cells, an aggressive Burkitt lymphoma cell line. As with the murine system, we tested all of the antibodies in three conditions: (i) monotherapy (antibodies alone), (ii) combination with anti-CD47, and (iii) combination with anti-CD20 (Fig. 1f–h). We identified functional antibodies that were consistent across all three treatment conditions (Supplementary Fig. 1e, Supplementary Fig. 2b, c). Of note, a strong association was observed with antibodies targeting MHC molecules on the lymphoma cells, including antibodies to MHC-I (HLA-A, B, C; β2M), and MHC-II (HLA-DR, DQ, DP; HLA-DR) components. Moreover, we identified antibodies targeting CXCR4, CD38, CD147, CD71, CD98 and CD45RA as top hits across multiple treatment conditions. Again, we observed a modest trend between the degree of antibody binding and elimination of the lymphoma cells in culture with some notable standouts (Fig. 1i–k). As an example, an anti-LILRB1 antibody had no substantial effect as a single agent but exhibited the greatest anti-tumor response in combination with anti-CD47 (Fig. 1i, j). This observation is consistent with the reported function of LILRB1, which is an inhibitory receptor expressed on macrophages that acts as an immune checkpoint^10^ and indicates this screening platform can be useful to identify antibodies that act by either opsonizing the lymphoma cells, exerting a functional effect directly on the macrophages, and/or cross-linking macrophages and lymphoma cells to promote phagocytosis.

### Antibody combinations to maximize macrophage-mediated cytotoxicity of B-cell lymphoma

Together, our murine and human studies identified several therapeutic targets and corresponding antibodies that worked alone or in combination with CD47- or CD20-targeting antibodies (Fig. 1l), and that could significantly stimulate macrophage-mediated cytotoxicity of lymphoma cells (Supplementary Fig. 2d, e). Notably, some hits—including CXCR4, CD47, CD147, CD98, and I-A/I-E (mouse) / HLA-DR, DP, DQ (human)—were consistently observed across both species (Fig. 1m). To confirm the clinical relevance of the identified hits, we validated their transcript expression using independent, publicly available RNA transcriptional datasets from DLBCL patient samples (Supplementary Fig. 3a, b). Surface expression was also confirmed for a panel of hits by flow cytometry on five lymphoma cell lines (Supplementary Fig. 3c, d). Since combinations of therapeutic antibodies have shown promise in ongoing clinical trials for patients with lymphoma, particularly combinations of anti-CD47 and anti-CD20 antibodies^17^, we next evaluated whether combinations of antibodies to the targets we identified could elicit more robust anti-tumor responses in vitro in the murine and human systems. Given the central importance of MHC-I molecules in regulating macrophage phagocytosis, we examined both wild-type and MHC-I knockout cell lines. Using syngeneic mouse macrophages and A20 lymphoma cells, we evaluated the combination of the top five antibodies from the murine screen—anti-CD24, CD95 (FAS), CD40, CXCR4, and CD79b—along with antibodies against CD47, CD20, and PD-1 as comparisons. We individually combined all of these for a total of 64 different antibody combinations to identify those that exhibit the greatest activity for stimulating macrophage anti-lymphoma functions (Supplementary Fig. 4a–c). We found that the A20 MHC-I knockout line was more vulnerable to ADCP, but otherwise similar trends were observed across the wild-type and MHC-I knockout lines. Additionally, we found that there were several antibody combinations that were substantially more effective than even the combination of anti-CD20 and anti-CD47. In particular, most combinations with anti-CD24 or anti-CD95 antibodies caused elimination of nearly all lymphoma cells from the co-cultures. These findings suggest that highly active antibody combinations can be identified that maximize the ability of macrophages to attack and eliminate lymphoma cells.

Similarly, we evaluated eleven antibody combinations in the human system. The combinations included antibody hits from the human screen (anti-B2M, CD38, LILRB1, and CD71), the mouse system (anti-CD24, CD79b and CD40), and shared antibodies (anti-CXCR4 and CD147), along with CD47- and CD20-targeting antibodies. We tested the antibodies in co-culture assays using wild-type Raji cells, Raji MHC-I KO cells, and Toledo cells (a human DLBCL cell line). From these efforts, we identified over a dozen antibody combinations that were highly active across all three cell lines tested. Among the most effective of these combinations was anti-CD47 combined with either anti-B2M, anti-LILRB1, anti-CD71, or anti-CD38 (Supplementary Fig. 4d). Together, these findings indicate that highly active combination therapies can be identified, and the effectiveness of these combinations may be comparable to or exceed that of anti-CD20 combinations. Furthermore, the MHC-I knockout line was generally more sensitive to macrophage-dependent killing in response to antibodies, again underscoring the importance of MHC-I molecules in protecting lymphoma cells from macrophage phagocytosis.

### Development of a rapid system to create and evaluate bispecific antibodies for macrophage-mediated cytotoxicity

**Fig. 2:**
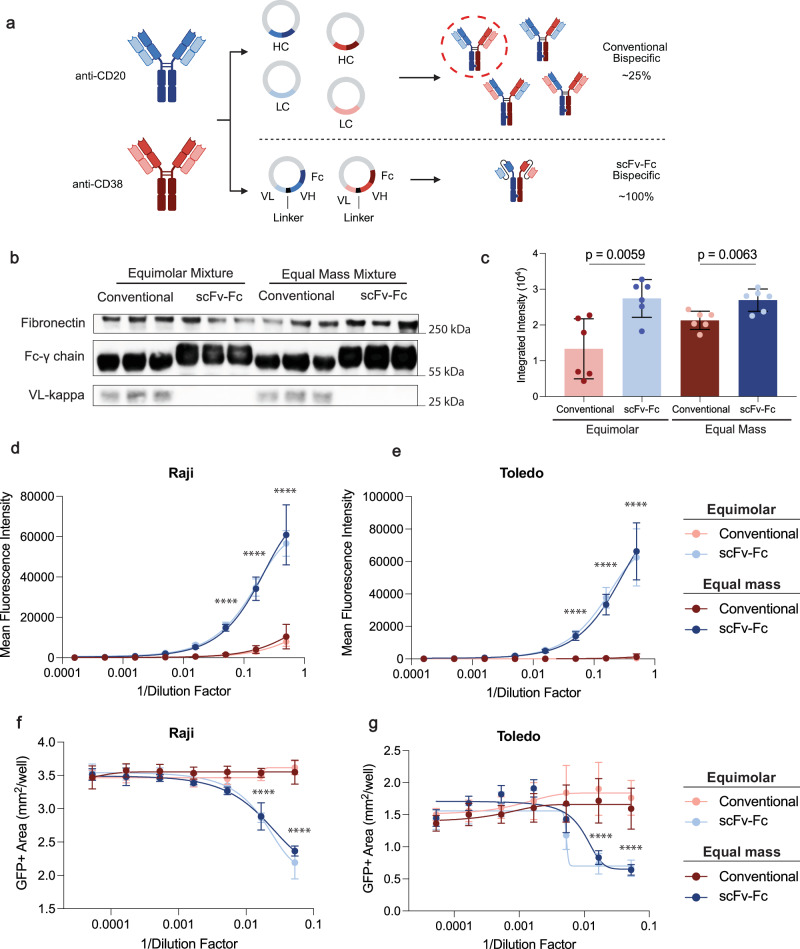
A heterodimeric scFv-Fc format enables high-throughput engineering and screening of bispecific antibodies. **a** Schematic representation of the synthesis of conventional bsAbs versus heterodimeric scFv-Fcs bsAbs. Conventional bsAbs were created by transfecting four plasmids containing the full-length heavy chain (HC) and light chain (LC) pairs, whereas scFv-Fcs were created by transfecting two plasmids, each encoding an scFv-Fc to an individual target. The conventional method can generate up to four different species arising from different light chain pairings, with only one out of four species being the correctly assembled bispecific. In contrast, the heterodimeric scFv-Fc approach generates one main functional species. Created in BioRender. Pagès-Geli, C. (2026) https://BioRender.com/g7ejm2o. **b** Western blot of the supernatant of cultures transfected with equimolar or equal mass mixtures of plasmids. Blots were stained with antibodies for the Fc-region, the kappa VL, and the loading control (fibronectin). One blot was performed to analyze Fc and VL, and two additional blots performed to quantify Fc of *n* = 6 biological replicates. **c** Quantification of the intensity of the Western blot Fc bands depicting a statistically significant difference in the antibody amount between the conventional and the scFv-Fc bsAb method. An unpaired two-tailed t-test was used to evaluate the difference between the groups. **d**,**e** Flow cytometry binding assays of the conventional bsAb or the scFv culture supernatants under different dilution factors to the indicated lymphoma cell lines (**d**, Raji; **e**, Toledo). **f**,**g** Co-culture assays using human macrophages were used to determine the potency of the different dilutions of the conventional bsAb or the scFv culture supernatants across the indicated lymphoma cell lines (**f**, Raji; **g**, Toledo). **c–g** Data represent mean ± SD from *n* = 6 individual transfection reactions. **d–g** Two-way ANOVA with Sidak adjustment for multiple comparisons was used to evaluate the difference between scFv-Fc and conventional formats with ****p < 0.0001. Source data are provided as a Source Data file.

Our unbiased screening efforts indicate that additional antigens exist on lymphoma cells that can be targeted to stimulate robust anti-tumor responses by macrophages, and that combinations of these antibodies could yield further benefits. We therefore reasoned that the best way to leverage the hits would be to create bsAbs, which could maximize immune responses in the tumor microenvironment while minimizing side effects. Thus, we sought to develop a high-throughput system to rapidly engineer and screen libraries of bsAbs targeting the hits from our screens. We hypothesized that a heterodimeric scFv-Fc format—single-chain variable fragments fused to human IgG1 Fc regions containing a knob or hole mutation^22^—could facilitate rapid assembly of bsAb libraries at scale—since they simplify cloning and eliminate the “light chain pairing issue” (Fig. 2a). To evaluate this possibility, we generated a CD20xCD38 bsAb via conventional methods and compared this to a CD20xCD38 scFv-bsAb. Compared to the conventional bsAb, we found that cultures producing a CD20xCD38 scFv-Fc bispecific yielded higher antibody titers (Fig. 2b, c), showed greater binding to lymphoma cells (Fig. 2d, e), and promoted more effective lymphoma cell killing at lower concentrations (Fig. 2f, g). Beyond these benefits, due to the fact that each binding arm is encoded by a single DNA sequence on a single plasmid, we recognized that the heterodimeric scFv-Fc format could enable high-throughput engineering, production, and evaluation of combinatorial libraries of bsAbs. We therefore used this approach to rapidly generate a total of 156 unique bispecific macrophage-activating antibodies and screened them for their ability to stimulate macrophage-mediated cytotoxicity against lymphoma cells.

**Fig. 3:**
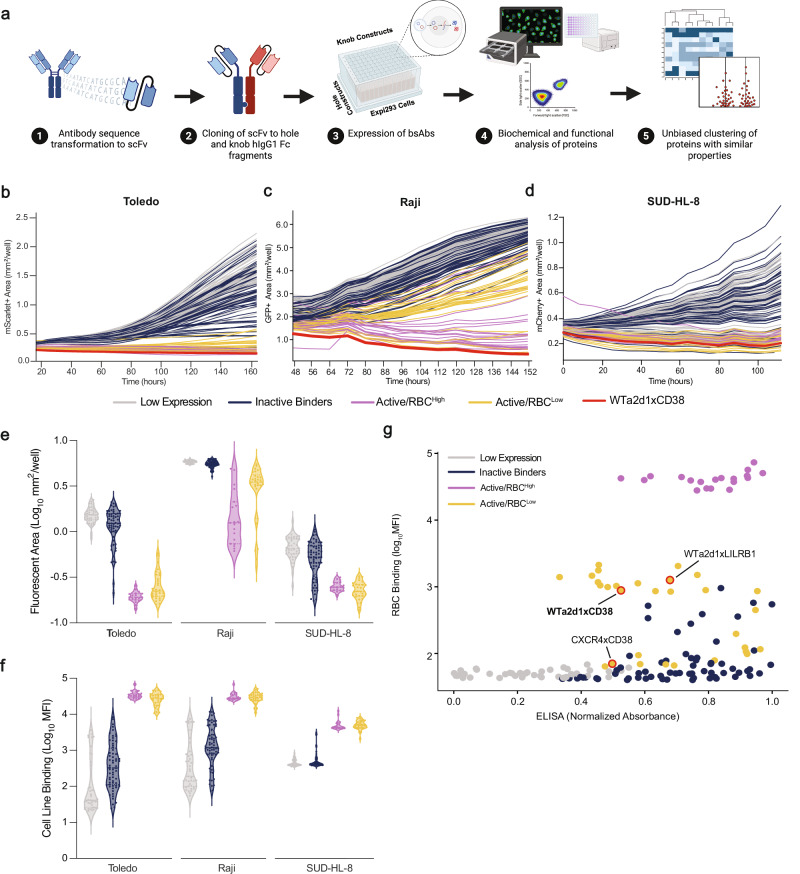
Combinatorial assembly of a bispecific antibody library to maximize macrophage-mediated destruction of human B-cell lymphoma. **a** Experimental approach for the generation and characterization of a combinatorial library of bsAbs targeting human B-cell lymphoma. scFvs were created based on publicly available antibody sequences, fused to modified human IgG1 Fc regions containing knob or hole mutations in individual plasmids, then plasmids were crossed and transfected into Expi293 cells to create a combinatorial library of bsAbs. The resulting bsAbs (*n* = 156) were tested for functional activity and binding to multiple lymphoma cell lines and RBCs. ELISA was performed to evaluate antibody expression. Finally, the properties of macrophage-mediated cytotoxicity, binding, and expression by ELISA were used for K-Means unbiased clustering of bsAbs with similar biochemical and functional properties. Created in BioRender. Pagès-Geli, C. (2026) https://BioRender.com/9dogq4b. **b–d** Growth of human lymphoma cells (b, mScarlet+ Toledo; c, GFP+ Raji; d, mCherry+ SUD-HL-8) in co-culture with human macrophages. Each curve depicts lymphoma growth in the presence of a different bsAb. Curves represent mean of three independent co-culture samples. Each color represents a different cluster of proteins based on functional properties. One bsAb selected for further evaluation (WTa2d1xCD38) is indicated in red. **e** Violin plots showing the log_10_-transformed fluorescent area upon co-culture with macrophages at the analysis time point for each lymphoma cell line and each bsAb cluster. Each point represents mean of three individual co-cultures. **f** Violin plot depicting binding of the antibodies to each lymphoma cell line based on clustering. Each point represents binding analyzed from one experiment. **g** Relationship between the different bsAb clusters with respect to binding to human RBCs and their expression by ELISA evaluated in one experiment for each parameter. Source data are provided as a Source Data file.

To begin, we curated a library of available antibody sequences to targets identified from our screens (Fig. 3a). We formatted these into scFvs, then fused them to a human IgG1 knob or hole Fc construct to facilitate heterodimerization^22^. Based on the results of our mouse and human screens, we targeted CD47, CD24, CD79b, CXCR4, CD40, CD95, CD38, CD71, LILRB1, and CD20. For targeting CD47, we tested two different single-domain binding modules: CV1 (high-affinity SIRPα decoy protein) and WTa2d1 (low-affinity SIRPα decoy protein)^15^. These variants were selected to evaluate differences in off-tumor toxicity that have been associated with high-affinity binding to CD47^23,24^. We also targeted PD-1 as a comparison. We crossed all of these binding arms with each other in two reciprocal formats (i.e., knob-hole and hole-knob), expressed them in Expi293F cells, and then evaluated the resulting antibodies for expression, binding, and function. We tested each bsAb in co-culture assays using human macrophages and three different human B-cell lymphoma cell lines: Raji, Toledo, and SUD-HL-8 (Fig. 3b–e). We also examined the expression of each bsAb by ELISA and their binding to each of the cell lines (Fig. 3f, g). Furthermore, since on-target red blood cell (RBC) toxicity has been observed with some anti-CD47 antibodies in clinical trials^17^, we also examined the ability of each bispecific to bind to human RBCs (Fig. 3g).

Across all of these assays, we found that four distinct categories of bispecific emerged from unsupervised clustering analysis: (i) Bispecifics that were highly active and exhibited minimal RBC binding (‘Active/RBC^Low^’), (ii) Bispecifics that were highly active and exhibited intense RBC binding (‘Active/RBC^High^’), (iii) Bispecifics with limited activity, variable lymphoma binding, and low or absent RBC binding (‘Inactive binders’), and (iv) Bispecifics that did not fold or express well (‘Low Expression’). Consistently, we found that bispecifics formulated with a CV1 binding arm exhibited exceptional anti-tumor effects but also exhibited strong binding to RBCs. Many bispecifics formulated with WTa2d1, CD20, or CD38 binding arms exhibited desirable properties of robust anti-lymphoma activity with minimal RBC binding. Other binding targets exhibited limited functional activity or did not express well (Fig. 3e–g). Across all three cell lines tested, we found that WTa2d1xCD38, WTa2d1xLILRB1, and CD38xCXCR4 bsAbs were consistently among the most effective agents for stimulating macrophage-mediated cytotoxicity of B-cell lymphoma cells and exhibited minimal or absent RBC binding (Fig. 3b–g). Overall, these findings indicate that these bispecifics may be ideal therapeutics for stimulating innate immune cells to attack and eliminate B-cell lymphoma.

### A WTa2d1xCD38 bispecific antibody is an optimal therapeutic candidate for B-cell lymphoma

**Fig. 4:**
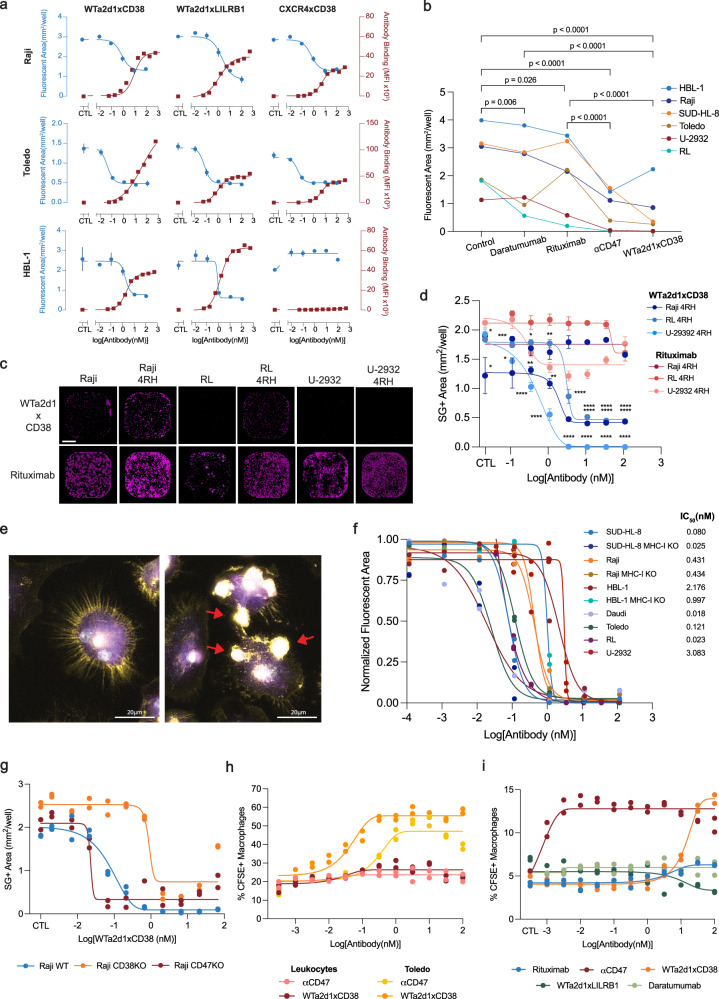
WTa2d1xCD38 stimulates maximal macrophage-mediated cytotoxicity of B-cell lymphomas. **a** Three purified bsAbs were evaluated for binding (red, right y-axis) and anti-lymphoma function (blue, left y-axis) using three human B-cell lymphoma lines. Curves represent titrations from three individual macrophage co-cultures (fluorescent area at 144 h, mean ± SD) or at least 3 technical replicates (antibody binding, mean). **b** Co-culture assays comparing the efficacy of WTa2d1xCD38 to the indicated therapies (10 µg/mL for each) at 144 h. Comparisons by two-way ANOVA with Tukey adjustment from 3 individual co-cultures. **c** Images from co-cultures of wild-type or rituximab-resistant (4RH) lymphoma lines treated with rituximab or WTa2d1xCD38 (10 µg/mL). Representative of three individual co-cultures per condition. Scale bar, 800 µm. **d** Co-cultures of human macrophages and rituximab-resistant (4RH) lines in response to WTa2d1xCD38 or rituximab. Curves represent titrations from three individual co-cultures. Fluorescent area normalized to maximum value for each cell line, data depict mean ± SEM compared by two-way ANOVA with Sidak adjustment. *p < 0.05, **p < 0.01, ***p < 0.001, ****p < 0.0001. **e** Immunofluorescence from co-cultures of human macrophages (cyan), mScarlet+ Toledo cells (magenta) and WTa2d1xCD38 (yellow) detected with Alexa 647 anti-human IgG. Representative of two independent experiments performed with similar conditions. Scale bar, 20 µm. **f** Co-cultures of human macrophages and ten human B-cell lymphoma lines, including wild-type and MHC-I KO variants, treated with WTa2d1xCD38. Curves represent titrations from ten individual co-cultures per condition. Fluorescent area normalized based on maximum value for each line. **g** Co-cultures of human macrophages and wild-type, CD38 KO, and CD47 KO Raji lymphoma variants treated with WTa2d1xCD38. Curves represent titrations from 2 individual co-cultures per condition. **h** Phagocytosis assays using CFSE-labeled Toledo cells or primary human leukocytes, each co-cultured separately with human macrophages in the presence of WTa2d1xCD38 or an anti-CD47 antibody at various concentrations. **i** Phagocytosis assays using CFSE-labeled RBCs co-cultured with human macrophages in the presence of the indicated antibodies. **h**,**i** Phagocytosis measured as percentage of CFSE+ macrophages. Points show measurements from at least 2 individual co-cultures per condition. Source data and exact *p* values are provided as a Source Data file.

We further investigated the three selected bsAbs—WTa2d1xCD38, WTa2d1xLILRB1, and CD38xCXCR4—for their ability to bind to lymphoma cells and stimulate macrophage-mediated cytotoxicity across a range of concentrations (Fig. 4a). All three bsAbs exhibited potent binding and efficacy for Raji and Toledo cells, whereas only the WTa2d1xCD38 and WTa2d1xLILRB1 were active against HBL-1 cells. Since the LILRB1-binding arm may primarily act by targeting human macrophages and is not known to cross-react with the mouse homolog, thereby limiting its use in mouse models, we selected the WTa2d1xCD38 for further characterization. Structural modeling of WTa2d1xCD38 confirmed the expected knob-into-hole association, and mass spectrometry showed correct bispecific assembly (Supplementary Fig. 5a, b). On-cell binding assays demonstrated that each arm binds its target antigen and that WTa2d1xCD38 displays high-affinity binding to both lymphoma cell lines and human-derived macrophages (Supplementary Fig. 5c–e).

To evaluate the ability of this antibody to elicit macrophage-mediated cytotoxicity, we performed additional co-culture assays with varying concentrations of the WTa2d1xCD38 bispecific. We found that the bispecific was more potent and more effective than both rituximab, a gold-standard clinical benchmark, and daratumumab, an FDA-approved anti-CD38 antibody, and it performed comparably to a monospecific anti-CD47 antibody (Fig. 4b, Supplementary Fig. 5e, and Supplementary Fig. 6a–i). Thus, the bsAbs may preserve the maximal activity of monospecific anti-CD47 agents while reducing the risks of hematologic toxicity. Moreover, since lymphoma cells can develop resistance to rituximab^25,26^, we also assessed the efficacy of WTa2d1xCD38 in human macrophage co-cultures with three different rituximab-resistant lymphoma cell lines. WTa2d1xCD38 remained highly active and significantly enhanced macrophage-mediated cytotoxicity of these cell lines, whereas responses were minimal or absent to rituximab or daratumumab (Fig. 4c, d and Supplementary Fig. 6d–f).

We next performed immunofluorescence analysis to further understand how the bsAb could mediate its effects. We found that the WTa2d1xCD38 bsAb could bind both macrophages and lymphoma cells. A subset of treated macrophages exhibited a distinct radial phenotype characterized by prominent microvilli protrusions, suggesting increased potential for immunological synapse formation^27,28^ (Fig. 4e, left and Supplementary Fig. 6j). Furthermore, immunofluorescent imaging revealed clear evidence of phagocytosis, with lymphoma cells observed within the cytoplasm of macrophages. We generally observed the greatest intensity of staining at the interface between macrophages and engulfed cancer cells, consistent with clustering and bridging of receptors at the interface to promote phagocytosis (Fig. 4e, right and Supplementary Fig. 6j).

We then evaluated the efficacy of the WTa2d1xCD38 bsAb across a broader panel of lymphoma cell lines, including MHC I-deficient lines (Fig. 4f). We individually tested ten different human lymphoma cell lines in co-culture assays using human macrophages and a range of concentrations of the WTa2d1xCD38 bsAb. Across all cell lines tested, the bsAb exhibited robust anti-lymphoma efficacy and potency, with IC_50_ values ranging as low as 18.0 pM (Daudi) to 3.08 nM (U-2932). The bsAb was generally more active for MHC-I deficient cell lines compared to their wild-type counterparts, resulting in ~2-3-fold improvements in the IC_50_ for SUD-HL-8 and HBL-1 variants, respectively.

To evaluate the importance of each individual target for the function of the WTa2d1xCD38 bsAb, we generated Raji cell variants in which CD38 or CD47 were knocked out. For each cell line, a titration of WTa2d1xCD38 was used to evaluate the potency of the antibody in co-cultures assays. We found that CD47 knockout had no substantial effect on the ability of the bsAb to elicit cytotoxicity—indicating that the bispecific can bind to CD38 alone, and demonstrating that genetic deletion of CD47 is equivalent to CD47 blockade in this system. On the other hand, the efficacy of the bsAb was preserved, but the potency was modestly reduced when tested with the CD38 knockout line (Fig. 4g). Together, these data indicate that monovalent binding to either target may be sufficient to promote macrophage responses, but the degree of response may be greatest when both targets are expressed on the surface of the target cells.

Since many monospecific CD47-targeting therapies have been limited by on-target toxicity to blood cells in clinical trials, we evaluated this risk using phagocytosis assays in which human macrophages were co-cultured with CFSE-labeled cells (Supplementary Fig. 7a). We first tested unfractionated human leukocytes and compared them to Toledo lymphoma cells. We treated the cells with varying concentrations of an anti-CD47 antibody or WTa2d1xCD38. We found that both antibodies resulted in minimal increases in leukocyte phagocytosis above background. However, WTa2d1xCD38 produced greater phagocytosis of Toledo cells than the anti-CD47 antibody across the dose titration (Fig. 4h). We also performed ex vivo assays with unfractionated PBMCs and the three validated bsAbs. We found that the bsAbs could bind to all major leukocyte subsets, with the greatest binding observed to B cells (Supplementary Fig. 7b, c). In a 24-hour depletion assay, we found that both WTa2d1xCD38 and CXCR4xCD38 could significantly deplete B cells to a degree that was comparable to rituximab, and WTa2d1xLILRB1 and CXCR4xCD38 showed some monocyte reduction that was not significant (Supplementary Fig. 7d). Interestingly, we did not observe any significant depletion of CD3 + T cells or NK cells. To further characterize the risk for hematological toxicity, we compared RBC phagocytosis in response to the WTa2d1xCD38, WTa2d1xLILRB1, rituximab, daratumumab, and anti-CD47. As expected, we found that the anti-CD47 antibody induced the greatest degree of macrophage engulfment of RBCs with an EC_50_ of 10 pM, whereas the potency of WTa2d1xCD38 to induce this effect was reduced by several orders of magnitude (Fig. 4i). WTa2d1xLILRB1, rituximab, and daratumumab all exhibited minimal RBC phagocytosis. Together, these findings indicate WTa2d1xCD38 and other bsAbs that we identified may exhibit a more favorable therapeutic index than conventional anti-CD47 antibodies, and that incorporating screening of non-malignant cells might reduce the risks of off-tumor toxicity.

### WTa2d1xCD38 can induce anti-tumor responses in aggressive models of B-cell lymphoma

In preparation for mouse xenograft studies, we evaluated binding of WTa2d1xCD38 to human versus mouse macrophages. We found that WTa2d1xCD38 exhibits greater binding to human macrophages than NSG mouse macrophages (Supplementary Fig. 8a), suggesting it may be more active in fully human systems. However, in co-culture assays, we found that WTa2d1xCD38 could still induce anti-tumor responses by NSG mouse macrophages against Raji cells (Supplementary Fig. 8b). These findings gave us confidence to test WTa2d1xCD38 in xenograft models of aggressive B-cell lymphoma using mice. We selected NSG mice, which lack T-, B-, and NK cells but contain myeloid cells capable of responding to macrophage-directed therapies^29^. Although this strain may have impaired immune responses, it has been considered a gold-standard model for evaluating CD47-directed therapies since the mice have an allele of SIRPα that cross-reacts with human CD47^30^.

**Fig. 5:**
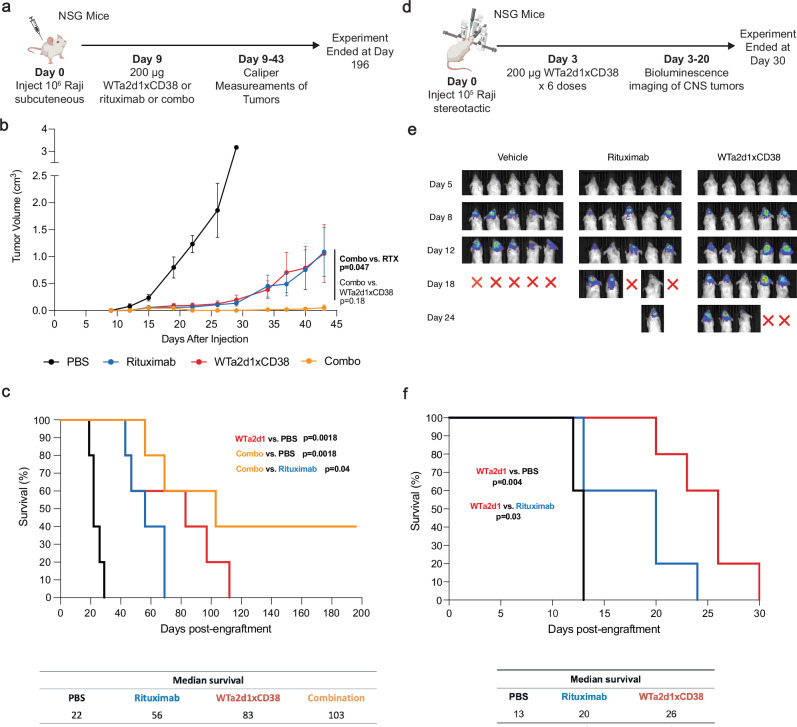
WTa2d1xCD38 exhibits anti-tumor efficacy in xenograft models of aggressive B-cell lymphoma. **a** Experimental setup of a xenograft experiment using Raji cells engrafted subcutaneously into the flanks of NSG mice. **b** Growth curves of Raji lymphoma tumors in NSG mice treated with the indicated therapies over time. Tumor growth was evaluated by caliper measurements. Mice were treated with control, 200 µg WTa2d1xCD38, 200 µg rituximab or the combination, with a total of seven injections (three times/week). Data are shown until day 43 post-tumor induction. Kruskal-Wallis test with Dunn’s adjustment for multiple comparisons was performed at day 43 to evaluate differences in tumor growth between conditions. **c** Survival analysis for the subcutaneous mouse model comparing the vehicle control, WTa2d1xCD38, rituximab, and combination treatment cohorts. Log-Rank test was used to evaluate the difference in the probability of survival between conditions. **d** Experimental setup of a CNS lymphoma xenograft model in which Raji cells were stereotactically engrafted into the brains of NSG mice. **e** Bioluminescence imaging using luciferase reporters of CNS tumors throughout the experimental period. Mice were treated with vehicle control, 200 µg WTa2d1xCD38 or 200 µg rituximab three times per week, with a total of six injections. **f** Survival analysis for the CNS lymphoma model comparing the vehicle control, WTa2d1xCD38 and rituximab treatment cohorts. Log-Rank test was used to evaluate the difference in the probability of survival between conditions. **b**,**c**,**d**,**f**, *n* = 5 mice per treatment cohort. **a**,**d** Created in BioRender. Pagès-Geli, C. (2026) https://BioRender.com/c1akt6e. Source data are provided as a Source Data file.

To understand whether WTa2d1xCD38 could exhibit therapeutic effects in vivo, we evaluated its efficacy in two xenograft models of aggressive B-cell lymphoma. First, we engrafted Raji cells subcutaneously into the flanks of NSG mice (Fig. 5a). After establishing tumors, we randomized the mice into four treatment groups: vehicle control, 200 µg WTa2d1xCD38, 200 µg rituximab as a standard-of-care benchmark, or the combination of both treatments. We found that tumor growth was dramatically inhibited by treatment with either WTa2d1xCD38 or rituximab compared to the vehicle control group. Importantly, the combination of the two antibodies resulted in a significant reduction in tumor burden compared to rituximab alone (Fig. 5b). In terms of survival, both WTa2d1xCD38 and rituximab significantly prolonged survival relative to control, with the combination group demonstrating the most pronounced survival benefit and was the only treatment cohort where mice were fully cured (Fig. 5c). In a second model, we stereotactically engrafted Raji cells into the brains of NSG mice as a model of primary central nervous system lymphoma (PCNSL), a highly aggressive extranodal B-cell lymphoma with few effective treatment options (Fig. 5d)^31^. We randomized mice to treatment with vehicle control, rituximab, or WTa2d1xCD38. Again, we found that WTa2d1xCD38 inhibited tumor growth and significantly prolonged survival compared to the control group and rituximab (Fig. 5e, f and Supplementary Fig. 9). Together, these findings indicate that WTa2d1xCD38 robustly stimulates anti-tumor responses and could be a highly active therapy for patients with aggressive B-cell lymphomas.

## Discussion

Monoclonal antibody therapies are among the most successful therapies for B-cell lymphomas and other hematologic malignancies^32^. These therapies work in part by activating innate immune cell effector functions, including macrophage ADCP. This function can be further enhanced by Fc engineering strategies or by blockade of the CD47/SIRPα macrophage immune checkpoint^15,33^. Here, we describe a rational strategy—deemed ‘tactical surfaceome profiling’—to identify targets of opsonization and antibodies that stimulate macrophage-mediated cytotoxicity of lymphoma cells. Our studies underscore the importance of MHC molecules in protecting lymphoma cells from macrophage attack. Furthermore, our screens identified additional checkpoint molecules, such as LILRB1 and CD24, that could be targeted for therapeutic purposes in B-cell lymphoma. Our screens also identified a multitude of unappreciated targets and antibodies that could be further advanced to develop additional therapies that stimulate macrophages to eradicate lymphoma cells. Interestingly, we also identified a number of cell surface targets that were highly expressed on B-cell lymphoma, yet their corresponding antibodies did not stimulate ADCP. These antigens may nonetheless be valuable targets for other therapeutic modalities, such as antibody-drug conjugates, CAR-T cell therapies, or bispecific T-cell engaging therapies.

A limitation of our screens is that they do not sample every possible antigen and every possible antibody that binds to the lymphoma cell surface. However, we provide comprehensive surfaceome profiling of B-cell lymphoma that is paired with functional anti-lymphoma responses by macrophages. From our efforts, we have identified consistent principles for macrophage-mediated cytotoxicity across mouse and human studies. Thus, the targets and hits we identified from our screening efforts are true positives that were validated by confirmatory studies in vitro. Furthermore, the results of our screening efforts and combinatorial studies successfully guided and informed the development of a compendium of highly active bsAbs.

Of note, we demonstrate the utility and feasibility of a high-throughput system to engineer and evaluate bsAbs. This strategy was enabled by the use of heterodimeric scFv-Fcs, which resulted in greater expression and activity than a conventional bsAb approach. These benefits are achieved since the heterodimeric scFv-Fc format eliminates the “light chain pairing issue” by constraining production to only one bispecific species with high purity. This is the key enabling feature of the heterodimeric scFv-Fc format that allows these molecules to be produced and tested functionally at scale. Moreover, the heterodimeric scFv-Fc format offers vast combinatorial potential such that similar libraries with thousands or millions of bsAbs could reasonably be produced and screened in the future. In this example, we used this system to create and discover bispecifics that maximally activate macrophage-mediated cytotoxicity of lymphoma cells while minimizing binding to RBCs. These properties can enhance the therapeutic index for targeting B-cell lymphoma by increasing specificity to the tumor microenvironment. Importantly, bispecifics with the most favorable properties could not be predicted based on their intended targets alone; instead, they were best identified by integration across multiple biochemical and functional studies. Thus, our strategy for rapid engineering and evaluation of bispecifics offers opportunities to advance the field of bsAb development. Furthermore, this approach can be applied to other types of cancer or other immune effector cells to yield highly active bsAbs that stimulate immune-mediated cytotoxicity.

Our studies help guide the best strategies to target the CD47/SIRPα axis for future drug development efforts. We found that targeting of CD47 may be best achieved using a bispecific format in which one arm comprises a low-affinity CD47-binding domain. This design may reduce the risk of hematologic toxicity while still maintaining robust anti-tumor responses by macrophages. Indeed, we found that in a bispecific format, both the high- and low-affinity CD47-binding domains could promote macrophage-mediated cytotoxicity. However, bsAbs containing WTa2d1 demonstrated significantly less binding to RBCs.

We also acknowledge the limitations of NSG xenograft models in our studies, which may overestimate the degree of response from CD47 blockade and limit our ability to evaluate toxicity. At the same time, the effects of our bsAbs are likely underrepresented in these models since they exhibit reduced or absent binding to mouse macrophages. Nevertheless, we believe the NSG studies are useful to show that the bsAbs are suitable with respect to their in vivo stability and pharmacokinetic properties to reach the tumor microenvironment and exhibit an anti-tumor effect. Ultimately, we believe that toxicology studies in non-human primates or early evaluation in clinical trials (e.g., Phase 0 studies) may be the most appropriate next step to determine the therapeutic potential for patients.

Among the most effective bsAbs that we generated were WTa2d1xCD38, WTa2d1xLILRB1, and CXCR4xCD38. These agents exhibited minimal RBC binding while robustly stimulating anti-lymphoma responses in vitro and in vivo. The WTa2d1xLILRB1 is of interest because it targets two known macrophage immune checkpoints^10^. CD38 is an established therapeutic target in multiple myeloma, and it is known to be expressed by B-cell lymphomas. However, prior trials examining the effects of the anti-CD38 antibody daratumumab observed limited single-agent activity for this drug in B-cell NHL^34^ despite its proven efficacy in multiple myeloma. Our studies suggest that the CD47/SIRPα macrophage immune checkpoint may be a significant barrier that prevents daratumumab from engaging macrophages as immune effector cells. The WTa2d1xCD38 bsAb that we developed may be an ideal way to maximize anti-lymphoma responses by macrophages in this context. Moreover, since macrophages also express CD38, it is possible that this bispecific is acting by multiple functions: (i) opsonizing cancer cells, (ii) blocking the CD47/SIRPα macrophage immune checkpoint, (iii) inhibiting CD38-mediated immunosuppression, and (iv) cross-linking macrophages and cancer cells to enhance immune effector functions (Supplementary Fig. 10). Since WTa2d1xCD38 exhibits minimal RBC binding, it may be an ideal agent to target macrophage-mediated killing of lymphoma cells while offering a maximal therapeutic window. Moreover, given the expression of CD38 on other hematologic malignancies, this therapy may be effective for other disease indications beyond B-cell lymphoma. Our findings indicate these therapeutics hold tremendous potential and should be translated to the clinic for further investigation.

Overall, our study highlights the potential of high-throughput functional screens to identify novel targets and therapies for inducing macrophage-mediated destruction of B-cell lymphoma. The development of a WTa2d1xCD38 bsAb holds promise as a therapeutic for patients since it effectively boosts macrophage anti-lymphoma activity while minimizing the risk of hematologic toxicity. Our findings reveal new opportunities for therapeutic intervention by enhancing the immune system’s innate capabilities, aiming for improved treatment outcomes for patients with aggressive B-cell lymphomas. Overall, our approach of ‘tactical surfaceome profiling’ provides a blueprint that can be rapidly applied to create new therapies for other types of cancers.

## Methods

Our research complies with all relevant ethical regulations and was conducted with approval from the biosafety committees of Whitehead Institute, Vall d’Hebron Institut de Recerca (VHIR), and Beth Israel Deaconess Medical Center (BIDMC). Animal studies were performed according to protocols approved by the MIT Committee on Animal Care (#2304000511) and the Comité de Ética de Experimentación Animal of the VHIR (CEEA 27/19). The secondary use of discarded blood products from de-identified donors was determined to be exempt from review by the MIT Committee on the Use of Humans as Experimental Subjects and the BIDMC Committee on Clinical Investigations.

### Cell lines and culture

A20 (BALB/c B-cell lymphoma) and Raji (human Burkitt lymphoma) cell lines were obtained from ATCC. Toledo (human DLBCL) and SU-DHL-4 (human DLBCL) cell lines were obtained from the Koch Institute High Throughput Services Core. SUD-HL-8 (human large B-cell lymphoma), HBL-1 (AIDS-related NHL), and Daudi (human Burkitt lymphoma) cells were provided by Catherine Wu’s laboratory (Dana-Farber Cancer Institute). Pairs of wild-type and rituximab-resistant lines were provided by Francisco J. Hernandez-Ilizaliturri’s lab (Roswell Park Cancer Institute). All cell lines were cultured in RPMI (Thermo Fisher) supplemented with 10% ultra-low IgG fetal bovine serum (FBS) (Thermo Fisher), 100 units/mL penicillin, 100 µg/mL streptomycin, and 292 µg/mL L-glutamine (Thermo Fisher). Cell lines were maintained in humidified incubators at 37 °C with 5% carbon dioxide.

### Generation of knockout cell lines

MHC-I knockout A20 and Raji variants were made by CRISPR-Cas9 genome editing using a Cas9 endonuclease as previously reported^35^. In brief, mouse TAP1 gRNA, human B2M gRNA, Cas9 endonuclease, and universal tracrRNA were obtained from Integrated DNA Technologies (IDT). The crRNA:tracrRNA complex was delivered by electroporation using the Neon Transfection System (Thermo Fisher). Cells were sorted for MHC-I as negative selection, and the sorted population was subsequently expanded.

To generate MHC-I knockout SUD-HL-8 and HBL-1 lines, Cas9-expressing variants were first created with lentivirus from the Broad Institute Genomic Perturbation Platform (GPP) protocol for low-throughput virus generation (https://portals.broadinstitute.org/gpp/public/resources/protocols) with the following modifications: 293 T cells (ATCC) were plated at 0.25 million cells/ml in 10 cm plates on day 0 to achieve a density of 70–80% prior to transfection with LentiCas9-blast (Addgene 52962), psPAX2 (Addgene 12260), and pMD2.G (Addgene 12259) using TransIT-LT1 (Mirius Bio). Virus was harvested in DMEM (Gibco) supplemented with 20% heat-inactivated FBS (Gibco), 5% MEM non-essential amino acids (Gibco), and 5% GlutaMAX (Gibco) at 24 and 48 h post-transfection, pooled, and concentrated using Lenti-X concentrator (Takara).

For viral transduction, 1.5 million cells of each cell line were plated in RPMI-1640 medium (Gibco) supplemented with 10% heat-inactivated fetal bovine serum (Gibco), 1% Penicillin-Streptomycin (Gibco), and 8 μg/ml polybrene (4 μg/ml for TMD8 cells, Sigma Aldrich) in a tissue culture-treated 12-well plate. Concentrated lentivirus was added to each well, spinfected at 800 x g for 2 h at 32 °C, and incubated overnight at 37 °C with 5% CO_2_. Transduced cells were washed with fresh RPMI medium and selected with blasticidin (Santa Cruz Biotech) on day 2 post-transduction.

MHC-I knockout lines were generated via delivery of sgRNA targeting B2M (sgRNA sequence: GGCCGAGAUGUCUCGCUCCG). B2M sgRNA was cloned into the lentiGuide-Puro vector (Addgene 52963) and transduced into the Cas9-expressing cell lines. Cells were selected with puromycin on day 2 post-transduction and validated with flow cytometry.

CD47 and CD38 knockout variants of Raji cells were generated by Synthego/EditCo as express knockout pools using a multiguide approach. The following gRNA sequences were used to target CD47: GAAGUCUCACAAUUACUAAA, CUUGUUUAGAGCUCCAUCAA, CUACUGAAGUAUACGUAAAG. The following gRNA sequences were used to target CD38: UGCGAGUUCAGCCCGGUGUC, UGCUCGCGGUGGUCGUCCCG, GACGGUCUCGGGAAAGCGCU. Knockout pool variants were confirmed to have 94% (CD47) and 100% (CD38) indel percentage by EditCo ICE analysis.

### Generation of fluorescent cell lines

StayGold+ cell lines were generated by transduction of cells with a lentivirus encoding hStayGold (Genbank LC593679.1) under the control of an EF1-ɑ promoter (Vectorbuilder) followed by selection with puromycin. GFP+ Raji cells were generated as described previously^36^. To generate a stable mScarlet Toledo line, a DNA fragment encoding the monomeric mScarlet red fluorescent protein was synthesized (IDT) and cloned into an AAVS1-homology donor vector previously published^37^ using HiFi DNA Assembly (NEB) following restriction with XbaI and MluI digest of the targeting donor vector and inclusion of homology arms on the synthesized mScarlet fragment. The AAVS1-mScarlet donor template vector was introduced in the cells along with an AAVS1-targeting Cas9 PX458 (#48138-Addgene) plasmid containing the sgRNA sequence: GGGGCCACTAGGGACAGGAT using nucleofection according to the manufacturer’s specifications (Lonza 4D Nucleofector, B-cell protocol) at a DNA mass ratio of 4:1 donor to Cas9 plasmid. The cells were selected using puromycin driven by the endogenous AAVS1 transcription via a splice acceptor and 2 A chysel sequence upstream of the puromycin cassette in the AAVS1-homology donor construct. Cells were then expanded and further enriched for reporter expression using fluorescence-assisted cell sorting. Cells were frozen immediately after FACS enrichment, banked, and thawed for use in experiments.

### Mouse macrophage derivation

Murine macrophages were derived from syngeneic BALB/c or NSG mice as previously described^38^. In brief, long bones from mice were collected, and bone marrow was mechanically extracted using a mortar and pestle. Cells were washed with PBS, then subjected to ACK lysis (ThermoFisher) to deplete RBCs. Unfractionated bone marrow cells were plated on Petri dishes (Corning) with 20 ng/mL murine M-CSF (Peprotech) for at least 7 days to differentiate into macrophages. Macrophages were washed, removed from plates using TrypLE (Invitrogen), and used for experiments or replated as necessary. Macrophages were generally used for experiments between days 7 and 21 of culture.

### Human macrophage derivation

Human macrophages were derived from peripheral blood monocytes of healthy human blood donors as previously described^38,39^. Briefly, leukocyte reduction collars from de-identified blood donors were obtained from the Crimson Core Biobank (Brigham and Women’s Hospital). Monocytes were isolated using StraightFrom Whole Blood CD14+ microbeads (Miltenyi) using an AutoMACS Pro or AutoMACS Neo Separator (Miltenyi). Purified monocytes were cultured in IMDM (ThermoFisher) + 10% low IgG FBS (ThermoFisher) + 1x penicillin, streptomycin, and glutamine (ThermoFisher) containing 20 ng/mL human M-CSF (Peprotech) for 7 days to differentiate into macrophages. Macrophages were generally used for experimentation between days 7 and 21 of culture and removed from plates by trypsinization and cell lifting before replating as necessary. Primary human specimens were consumed in our study and are not available for distribution.

### Long-term co-culture assays

Long-term co-culture of murine or human macrophages and lymphoma cells was performed using fluorescently labeled target cells as previously described^38^. Phenol-red free IMDM (Thermo Fisher) supplemented with 10% ultra-low IgG fetal bovine serum, 100 units/mL penicillin, 10 μg/mL streptomycin, and 292 μg/mL L-glutamine, and 20 ng/mL M-CSF was used. For antibody library screens, Lyoplate Mouse and Lyoplate Human Cell Surface Marker Screening Panel plates (BD Biosciences) were used. Library plates containing lyophilized antibodies were centrifuged at 300 x g for 5 min before reconstitution with 140 μl of IMDM using an ASSIST PLUS pipetting robot (Integra). To set up co-cultures, 20 μl containing 1 × 10^4^ macrophages and 2 × 10^3^ target cells (4:1 E:T ratio) were added to each well of a 384-well plate using an ASSIST PLUS. Subsequently, 20 μl from each Lyoplate well was added to achieve a final working concentration of 6.55 μg/ml. Finally, an additional 20 μl medium alone or medium containing anti-CD47 (anti-mouse clone MIAP410, anti-human clone B6H12) or anti-CD20 (anti-mouse clone MB20-11, anti-human rituximab biosimilar) was added to the wells. This achieved a final working concentration of 10 μg/mL of anti-CD47 or anti-CD20 as appropriate. For bispecific antibody libraries, 20 μl of supernatant was added to the wells containing effector and target cells. Cells were then co-cultured for up to 7 days with whole-well imaging of phase contrast and green and/or red channels performed every 8 hours using an Incucyte S3 Live Cell Imaging System (Sartorius). Automated imaging analysis was performed using Incucyte Analysis Software v2018B-2025A (Sartorius). Statistical comparisons between treatment conditions were typically performed at the last analysis time point or as otherwise indicated (112–168 h). Analysis of bsAb library performed at 164 h (Toledo), 168 h (Raji), and 112 h (SUD-HL-8). Monoclonal antibody treatments were typically used at working concentrations of 10 μg/mL except as otherwise indicated.

### Quantitative FACS-based surfaceome profiling using arrayed antibody libraries

To evaluate specific binding interactions, 1 × 10^5^ target cells were added in 100 μl to each well of the Lyoplate plate containing 20 μl of reconstituted, purified antibody for working concentration of 3.275 μg/mL. The plate was then incubated on ice for 30 min to facilitate binding. For detection of murine library binding, biotinylated goat anti-mouse, anti-rat, and anti-Syrian hamster antibodies, at a concentration of 1.25 μg/mL each, were added at 100 μl per well, while biotinylated anti-Armenian hamster antibody, at a concentration of 0.6 μg/mL, was added at the same volume, to each corresponding well. The plate was then incubated for an additional 30 min on ice. After washing, plates were incubated with Alexa Fluor 647 streptavidin, diluted 1:4000, to achieve a final concentration of 0.5 μg/mL for 30 min on ice. For human binding analysis, Alexa Fluor 647 Goat anti-mouse Ig and Goat anti-Rat Ig (BD) were added after primary antibody in each according well (dilution 1:200), following the same incubation times. Following the same steps, for bsAb binding, 20 μl of supernatant or purified antibody was incubated with cells for 30 min on ice and a secondary antibody AffiniPure Goat Anti-Human IgG, Fcγ fragment specific (Jackson ImmunoResearch, dilution 1:500) was used. Cells were then resuspended with 100 μl of FACS Staining Buffer (BD) for flow cytometry analysis, performed using a BD LSR Fortessa flow cytometer. The mean fluorescence intensity (MFI) was measured. Data analysis was conducted using FlowJo (version 10.9).

### ScFv library and plasmid generation

Antibody sequences were obtained from publicly available sources and were directed against the following targets: CD20 (rituximab, IMGT 161), LILRB1 (CD85) (US Patent Application Pub. No.: 20230235055A1), CD71 (delpacibart, IMGT 1374), CD38 (daratumumab, IMGT 301), CD40 (dacetuzumab, IMGT 232), CD47 (CV1)^15^, CD47 (WTa2d1)^15^, CD24-1^40^, CD24-2 (US Patent Application Pub. No.: 20210213055A1), CXCR4 (CD184) (ulocuplumab, IMGT 483), PD-1 (nivolumab, KEGG D10316), CD79b (U.S. patent no: 8,545,850 B2), and CD95 (U.S. patent no: 10,590,202 B2). The sequences were numbered using the Kabat numbering system and their VH and VL regions were fused through a (GGGGS)x3 linker. The sequence was reverse-translated and optimized for the *Homo sapiens* codon usage table. Gene blocks ordered from Twist Biosciences containing these sequences were engineered with flaking 5’ and 3’ multiple cloning sites containing EcoRI/BamHI and NotI. The cloning into IgG1-Knob or IgG1-Hole (knob: T270Y, T311Y; hole: Y396T, Y437T) vectors for fusion and expression was carried out using restriction enzyme digestion followed by T4 DNA ligation and bacterial transformation. All constructs were generated as IgG1 Knob or Hole fusions except WTa2d1, which was generated only as a Knob construct. Individual colonies were subjected to Nanopore sequencing (Quintara Biosciences) to verify correct gene insertion. The ZymoPURE Plasmid Miniprep Kit (endotoxin levels ≤1 EU/µg of plasmid DNA) was used to extract plasmid DNA from transformed bacteria, which was subsequently used for transfection.

### Expression and purification of recombinant antibodies

For the comparison between a conventional bsAb and a scFv-Fc, two pairs of heavy chain and light chain plasmids or purified pairs of scFv-IgG1-Knob and scFv-IgG1-Hole plasmids were co-transfected at equimolar (330 fmol of total DNA) or same amount of DNA (1 μg per construct) and transfected into 900 μl of Expi293F cells at 3 × 10^6^ cells/mL (ThermoFisher) in 96 deep-well plate (USA Scientific) with ExpiFectamine 293 Transfection Kit (ThermoFisher) following the manufacturer’s recommendation and incubated at 37 °C, 8% CO2 with shaking at 900 rpm for 7 days. In other circumstances, purified pairs of IgG1-Knob and IgG1-Hole plasmids were co-transfected to a final concentration of 1 µg/mL of each into 900 μl of Expi293F cells. Cells were pelleted at 3000 x g for 20 min. The antibody supernatants were diluted two-fold with PBS and used for further analysis.

### ELISA

Immulon 4HBX Flat Bottom plates (ThermoFisher) were coated with 1:1000 dilution in PBS of anti-human IgG Fc (Jackson Immunoresearch) overnight at 4 °C. The following day, the plate was washed three times with PBST and blocked with 1% BSA in PBST for one hour at room temperature. The plate was then washed with PBST, loaded with the diluted transformed supernatants from Expi293F cells, and incubated for another hour at room temperature. Upon washing again, anti-human IgG H + L (Jackson Immunoresearch) was added to the plate at 1:100,000 dilution in 2% (w/v) non-fat dry milk in PBST and incubated for another hour at room temperature. Finally, another set of washes was performed and 1-Step Turbo TMB Elisa Substrate (ThermoFisher) was added to the plate and the reaction was quenched with 10% phosphoric acid after 3 min. The absorbance was assessed at 450 nm.

### Western blot

SDS-PAGE and transfer were adapted from ThermoFisher’s protocol. Briefly, equivolumetric amounts of supernatant from Expi293 transfected cultures were mixed with NuPAGE LDS Sample Buffer and NuPAGE Reducing Agent (ThermoFisher), heated at 95 °C for 5 min, then loaded onto NuPAGE Bis-Tris (4–12%) Mini Protein Gels (ThermoFisher). Electrophoresis was conducted at 150 V for 30 min in a mini-gel tank (ThermoFisher) with MES SDS Running Buffer (ThermoFisher). The gel was transferred to a PVDF membrane (ThermoFisher) using the same mini-gel tank with the NuPAGE Transfer Buffer (ThermoFisher) for 1 h at 50 V. Gel blotting was modified from a previous protocol^41^. Briefly, the membrane was washed twice with 1% (v/v) TBS-Tween-20 (TBS-T), then blocked overnight at 4 °C with 5% (w/v) nonfat dry milk in TBS-T with a 1:1000 dilution of anti-fibronectin antibody (Invitrogen, clone: FN-3) as a loading control. On the next day, the membrane was washed twice with TBS-T and incubated for 60 min at RT with a 1:5000 dilution of the secondary antibodies: HRP anti-human Fc γ-chain specific (Jackson Immunoresearch), HRP anti-human VL-Kappa (Invitrogen, A18853), and HRP anti-mouse IgG Fc γ-chain specific (Jackson Immunoresearch) in blocking buffer. Finally, the membrane was washed twice with TBS-T and developed with an ECL substrate (ThermoFisher). For measurements, this protocol was performed without VL-Kappa staining, and only the predominant human Fc band was subjected to quantification. Luminescence images were acquired using the Azure 200 gel imaging machine, and the integrated intensity of the human Fc band was analyzed using ImageJ version 1.54p.

### Phagocytosis assays

In vitro phagocytosis assays were performed as previously described^15^. CSFE-labeled leukocytes, Toledo cells, or RBCs were used as target cells. RBCs and unfractionated leukocytes were purified from discarded blood specimens from healthy human donors. Live cells were collected, washed, and co-cultured with human macrophages at a target macrophage ratio of 4:1. Cells were co-cultured with a log-titration of antibodies starting at 0.1 mg/ml or in the presence or absence of different antibodies at a final concentration of 10 µg/mL. Cells were co-cultured for 2 h in serum-free IMDM in round-bottom ultra-low attachment 96-well plates (Corning). After the incubation period, cells were washed and analyzed by flow cytometry. Macrophages were identified using APC anti-SIRPα (BioLegend) or APC anti-CD163 (Biolegend) and target cells were identified by CFSE fluorescence. Phagocytosis was quantified as the percentage of macrophages that contained CFSE signal. Phagocytosis was normalized to the maximal response. Dose-response curves were generated using Prism version 9.2.0 (GraphPad).

### Recombinant protein production and purification

WTa2d1xCD38 plasmids were produced using ZymoPure MaxiPrep kit following manufacturer instructions. Plasmid concentration and purity were assessed using Nanodrop (ThermoFisher) and 1 µg/mL of each was employed to transiently transfect Expi293F cells (ThermoFisher) following manufacturer instructions. Subsequently, the culture was pelleted at 15,000 x g for 15 min and the supernatant was loaded in a Protein A column (Cytiva) equilibrated with TBS (pH 7.2). The column was washed with 25 ml of TBS and the protein was eluted with 100 mM glycine and 100 mM NaCl solution (pH 3.0). The eluate was brought to physiological pH with 1 M Tris (pH 8.0) and the solution was buffer exchanged to PBS using either dialysis bags (ThermoFisher) or spin-columns (Cytiva). Protein concentration was obtained using a Nanodrop (ThermoFisher) by taking the absorbance at 280 nm.

### Mass spectrometric analysis

Intact mass analysis of the protein samples was performed at the MIT Biopolymers & Proteomics Core. Protein samples originally in PBS buffer at a concentration of 1 µM were prepared for analysis (*n* = 2 biological replicates, no controls were included). For matrix crystallization, 1 µL of the sample was mixed with 1 µL of a sinapinic acid matrix solution (20 mg/mL in 70% acetonitrile and 0.1% trifluoroacetic acid) and spotted onto the target plate. Mass spectra were acquired using a Bruker microflex MALDI-TOF mass spectrometer operating in linear mode. Ions were accelerated with an ion source 1 voltage of 20 kV and an ion source 2 voltage of 18 kV, with the ion source lens voltage maintained at 9 kV. Pulsed ion extraction (PIE) was utilized with a delay time of 600 ns. Desorption and ionization were achieved using a laser operating at a repetition rate of 60 Hz, and each spectrum represented the accumulation of 300 laser shots. The linear detector voltage was set to 3.079 kV. Spectra were acquired and processed using Bruker Daltonics flexAnalysis software version 3.3.80.0.

### Immunofluorescence

Human monocyte-derived macrophages were differentiated and stained with CellTracker Green BODIPY (ThermoFisher) according to the manufacturer’s instructions. Stained macrophages were seeded at 100k/well into 8-well chamber slides (ThermoFisher) and incubated overnight at 37 °C in 5% CO_2_. The following day, mScarlet+ Toledo cells and WTa2d1xCD38 were added to each well at a final concentration of 100k/well and 1 µg/mL, respectively. Co-cultures were incubated for 30 min at 37 °C in 5% CO_2_. At the end of the incubation, the supernatant was carefully aspirated, and cells were fixed with 4% paraformaldehyde (PFA) in PBS (ThermoFisher) for 15 minutes at room temperature. Following fixation, the chamber slides were disassembled, and the slides were washed three times with ice-cold autoMACS Running Buffer (Miltenyi). Slides were then incubated in autoMACS running buffer with 0.5% (v/v) Tween-20 for 30 min at RT, washed and incubated overnight at 4 °C in running buffer. For antibody staining, slides were incubated with anti-human Alexa Fluor 647 IgG Fc γ-chain specific secondary antibody (Jackson ImmunoResearch), diluted 1:500 in autoMACS Running Buffer for 1 hour at RT in the dark. Slides were then washed three times with autoMACS Running Buffer. Finally, slides were mounted using ProLong Gold Antifade Mountant with DAPI (ThermoFisher) and cured overnight at RT in the dark. Images were acquired using a Keyence BZ-X810 fluorescence microscope equipped with a 40x objective lens and analyzed using BZ-X Viewer/BZ-X Analyzer software.

### Ex vivo depletion assay

PBMCs were isolated by Ficoll density gradient from peripheral blood of healthy donors. A total of 2×10^5^ cells were incubated for 24 h at 37 °C with rituximab, anti-CD47 (clone B6H12), daratumumab, WTa2d1xCD38, WTa2d1xLILRB1, or CXCR4xCD38 antibodies at 10 μg/mL. An untreated condition was included as a control. After 24 h, cells were stained with antibodies against CD19, CD3, CD14, and CD56 and analyzed by flow cytometry using a Beckman CytoFLEX instrument.

### Xenograft mouse models

NSG mice were obtained from Jackson labs (Strain: #005557) and used for experiments when approximately 6–12 weeks of age. Age and sex-matched mice were engrafted subcutaneously with 1 × 10^6^ GFP+ Raji cells in 50% (v/v) Matrigel Matrix (Corning) and PBS. Mice were randomized to treatment cohorts and then subjected to intraperitoneal treatment with vehicle control, 200 µg WTa2d1xCD38, 200 µg rituximab biosimilar (BioXCell), or the combination of both. Mice were treated 3 times per week with a total of seven injections. Tumor dimensions were measured by caliper twice per week and used to calculate tumor volumes using an ellipsoid formula: *length* ⋅ *width*^2^ ⋅ *π*/6. Mice were monitored for endpoint-free survival and euthanized based on humane experimental endpoints, including tumor burden >1.9 cm in diameter, body condition score <2, tumor ulceration, or tumor impeding eating, urination, defecation, or ambulation. Mice were independently monitored by veterinary staff to ensure endpoint criteria were not exceeded.

Eight-week-old female NSG mice (Charles River Labs, Strain: #614) were used to develop a model of PCNSL. Mice were initially anesthetized with isoflurane (1-2%), followed by intradermal injection of ketamine (80–100 mg/kg) and xylazine (5–10 mg/kg) for anesthesia maintenance and analgesia. A total of 10^5^ luciferase+ Raji cells in 5 µl of PBS were injected intracerebrally using a Hamilton syringe with a 26-gauge needle at a rate of 1 µl/min, guided by stereotactic coordinates (1 mm anterior, 1.8 mm lateral right to the bregma, and 2.5 mm deep from the dura) on a Stoelting Just For Mice platform. Mice were then randomized into treatment groups and received intravenous injections of either vehicle control or 200 µg of WTa2d1xCD38 starting on day 3 post tumor injection, administered three times per week for a total of six injections. Tumor growth was monitored by bioluminescence imaging (BLI) using an IVIS Spectrum system (PerkinElmer) twice a week starting on day 5 post-intracerebral injection. Tumoral size was analyzed and quantified using Living Image software version 4.7.4 (PerkinElmer), and the total photons per second (ph/s) were recorded and analyzed. Mice were euthanized when humane experimental endpoint criteria were met, including appearance of neurological signs (hind limb paresis, ataxia, seizures, or loss of righting reflex), cranial swelling, poor general body condition (hunched posture, piloerection, or reduced responsiveness), and/or body weight loss of ≥20% from baseline.

### Statistical analysis

GraphPad Prism (versions 9.2.0, 10.2, and 11) were generally used to perform correlation, grouped analyses, and survival analyses. For exploratory statistical analysis of the arrayed antibody screens, one-tailed Student’s t-tests were performed with comparison between the mean of each antibody with the mean of the total population of all antibodies, and hits were further defined based on the 95th percentile threshold for fluorescent area (Graphpad Prism, Excel version 16.109.1). This approach aimed to detect decreases in tumoral area, indicating antibody activity, while increases reflected lack of effect with tumor areas reaching confluency. Volcano plots were generated using R (version 4.5.2) and RStudio (version 2025.09.2 + 418). Other comparisons between two groups were made with unpaired two-tailed t-tests (GraphPad Prism). For larger grouped analyses, two-way ANOVA was performed with correction for multiple comparisons as indicated. Survival analysis was performed using the log-rank (Mantel-Cox) test. For correlation between CD38 expression and anti-tumor function in co-culture assays, correlation analysis was performed using Pearson’s R (GraphPad Prism). Statistically significant differences were considered to be *p* < 0.05. Results are represented as mean ± SD or SEM as indicated. For statistical analysis of bsAb clusters, Python (v.3.10) was used. The datasets were normalized using the sklearn MinMaxScaler function, and Seaborn was used to calculate Euclidean distance and perform hierarchical clustering of the different therapeutic combinations in the human system. For K-means clustering, the dataset was first normalized using sklearn's StandardScaler (z-score normalization) and then fitted using sklearn’s K-means clustering function. The silhouette method was used to identify the optimal number of clusters. In some experiments, a limited number of replicates were excluded due to technical errors as indicated in the Source Data file, but they did not limit data interpretation.

### Reporting summary

Further information on research design is available in the Nature Portfolio Reporting Summary linked to this article.

## Supplementary information

Supplementary Information

Reporting Summary

Transparent Peer Review file

## Source data

Source Data

## Data Availability

Source data for intact mass analysis can be accessed via the MassIVE repository (10.25345/C5KW57Z41) with Dataset Identifier MSV000102419. The publicly available RNA transcriptional data used in this study are available in the Gene Expression Omnibus database under accession code GSE98588 and GSE147115. The remaining data supporting the results in this study are available within the paper, its Supplementary Information and Source data. Source data are provided with this paper.
