# Supplementary material for: High-throughput engineering of bispecific antibodies to enhance macrophage-mediated cytotoxicity of B-cell lymphoma
Source: Nat Commun. 2026 Aug 1;17:8491. doi: 10.1038/s41467-026-76180-5 (PMC13482096; doi:10.1038/s41467-026-76180-5)
Supplement: Supplementary file 1 [file 41467_2026_76180_MOESM1_ESM.pdf]

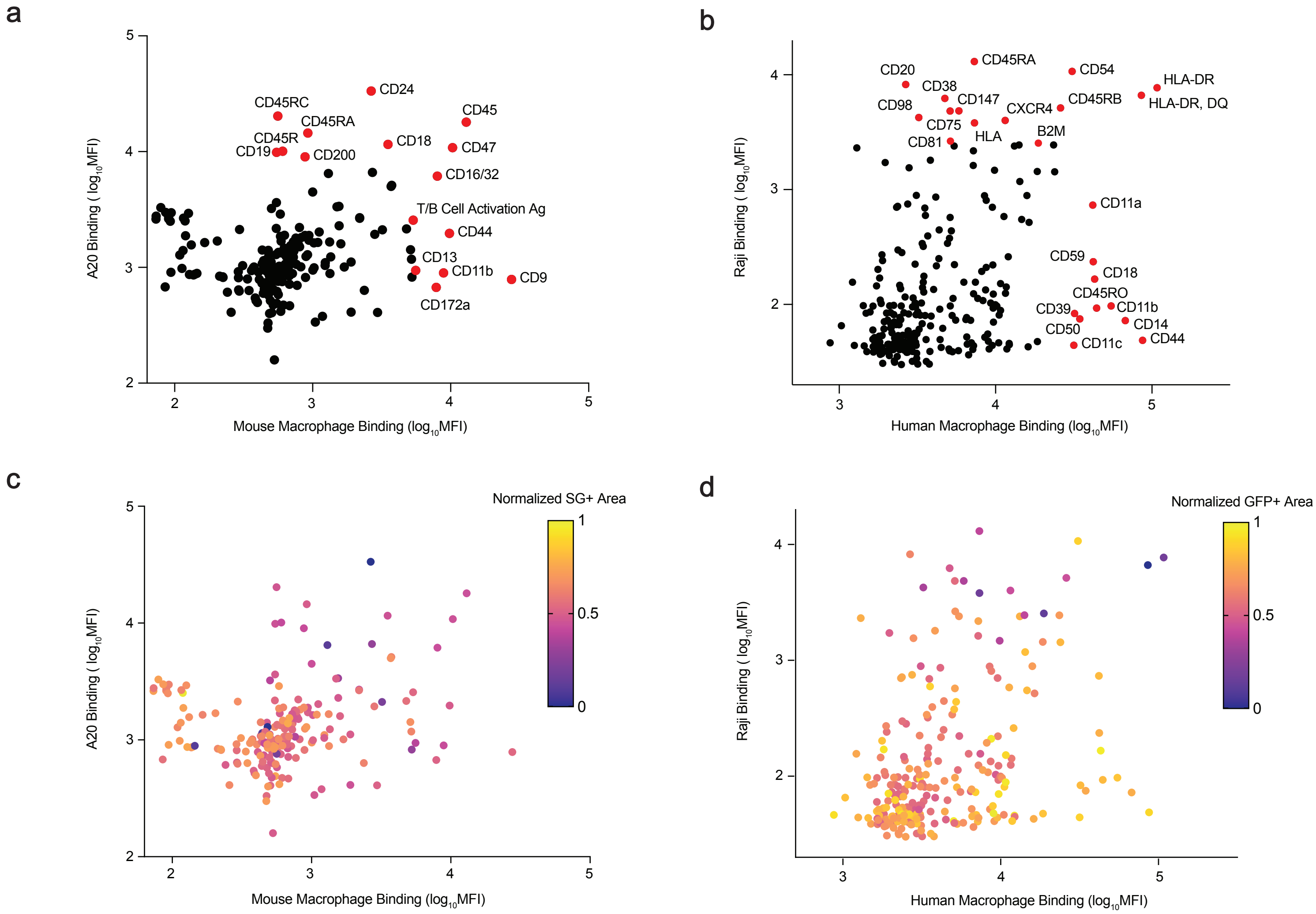

e

Mouse			Human		
**Alone**	**+αCD47**	**+αCD20**	**Alone**	**+αCD47**	**+αCD20**
CD24	CD24	CD24	HLA-A,B,C	HLA-A,B,C	HLA-A,B,C
CD95	CD95	CD95	HLA-DR,DP,DQ	HLA-DR,DP,DQ	HLA-DR,DP,DQ
CXCR4	CXCR4	CXCR4	HLA-DR	HLA-DR	HLA-DR
CD40	CD40	CD40	B2M	B2M	B2M
CD79b	CD79b	CD79b	CD98	CXCR4	LILRB1
CD53	CD53	I-A/I-E	CD147	LILRB1	CD38
CD81	I-A/I-E	Ly6A/E	CD71	CD98	CD47
CD98	Ly6A/E	CD47		CD147	CD71
CD147	CD11b	4-1BBL		CD45RA	CD37
I-A/I-E		CD200		CD45RB	CD22
				CD38	CD27
				CD49f	CD117
					CD128b

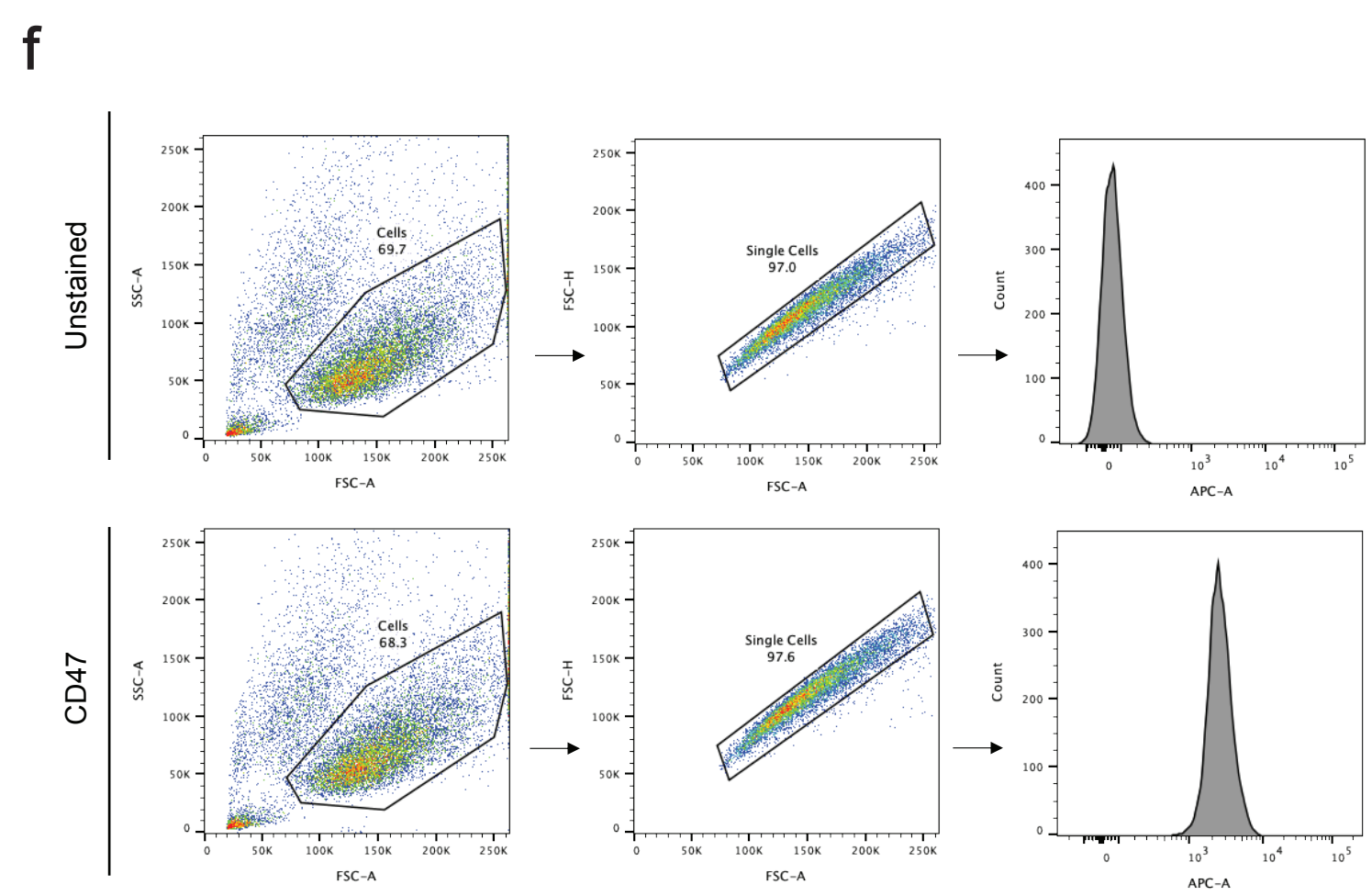

Supplementary Fig. 1 | Binding of antibodies from libraries to B-cell lymphoma cells and macrophages. a,b, Scatter plots depicting binding of individual antibodies from the murine antibody library to A20 lymphoma cells and mouse macrophages (**a**), and from the human antibody library to Raji lymphoma cells and human macrophages combined from n = 3 independent donors (**b**). Mean fluorescence intensity (MFI) was assessed by flow cytometry. The target antigens highlighted exceeded the 95th percentile for binding to cancer or macrophage cells. **c,d,** Multiple variable scatter plot depicting the relationship between the binding of the antibody library to lymphoma cells and/or macrophages and the functional anti-tumor effects of each antibody in co-culture with macrophages (color scale) in the murine (**c**) and human (**d**) systems as monotherapy. The color scale represents the cancer cell area at the last timepoint in co-culture assays (152 h), with blue indicating greater anti-lymphoma activity and yellow indicating lesser anti-lymphoma activity. Data represent mean of 2 individual co-cultures for function and one sample for binding for each antibody. **e,** List of antibodies defined as hits with fold-change thresholds set at the 95th percentile and p-values less than 0.05 and for each system and treatment condition. **f,** Representative plots showing general gating strategy used for MFI analysis (top, control; bottom, anti-CD47). **a-e,** Data derive from screens presented in Fig. 1. Source data are provided as a Source Data file.

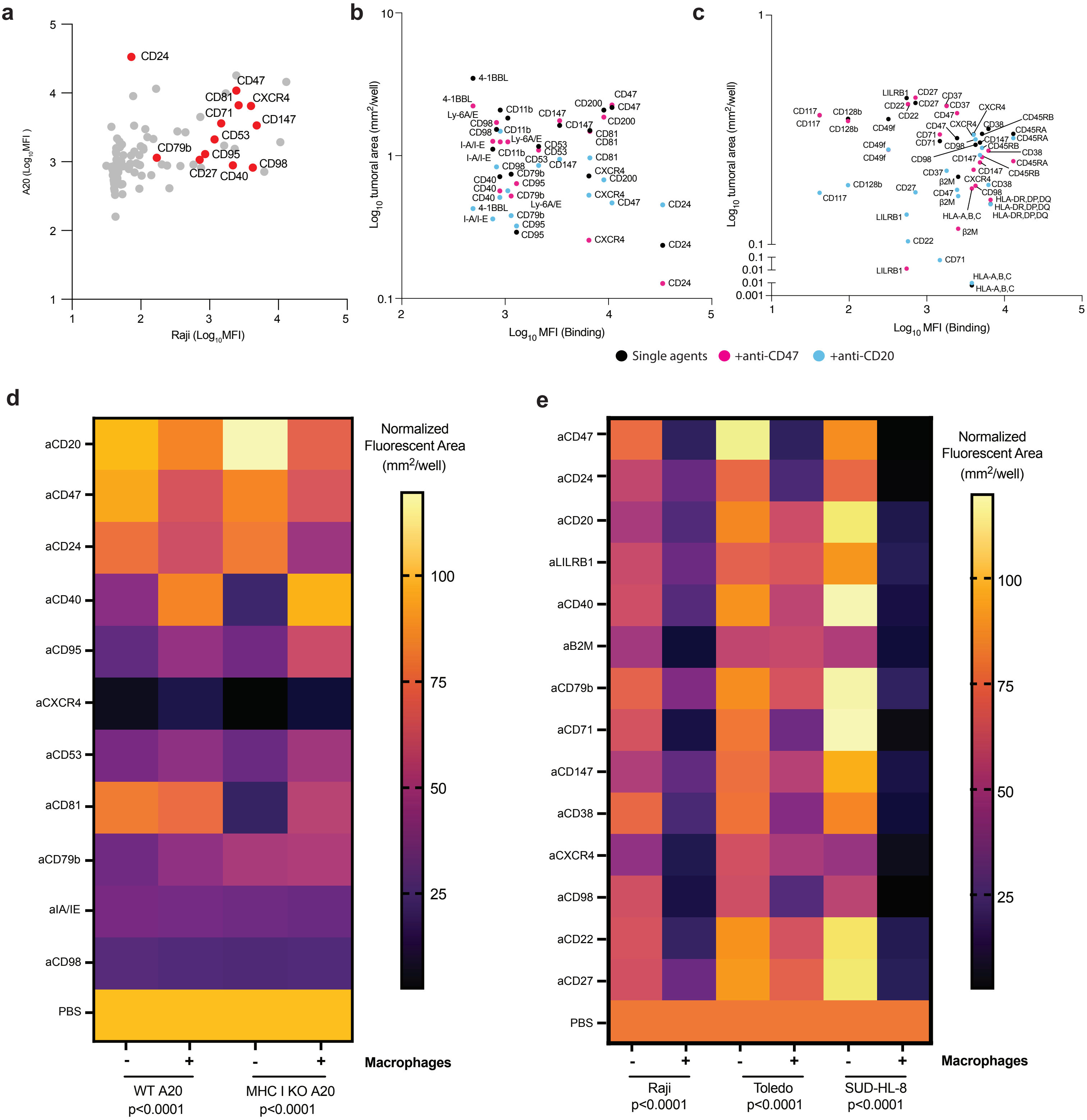
a
A20 (Log10MFI)
Raji (Log10MFI)
CD24
CD47
CD81
CXCR4
CD71
CD147
CD53
CD79b
CD95
CD98
CD27
CD40
b
Log10 tumoral area (mm2/well)
Log10 MFI (Binding)
4-1BBL
Ly-6A/E
CD11b
CD98
I-A/I-E
CD53
CD79b
CD40
CD95
CD147
CD200
CD47
CD81
CXCR4
CD24
c
Log10 tumoral area (mm2/well)
Log10 MFI (Binding)
LILRB1
CD27
CD37
CD117
CD128b
CD22
CXCR4
CD45RB
CD49f
CD47
CD38
CD45RA
CD71
CD98
CD147
β2M
HLA-DR,DP,DQ
HLA-A,B,C
Single agents
+anti-CD47
+anti-CD20
d
aCD20
aCD47
aCD24
aCD40
aCD95
aCXCR4
aCD53
aCD81
aCD79b
aIA/IE
aCD98
PBS
Normalized Fluorescent Area (mm2/well)
100
75
50
25
Macrophages
WT A20 p<0.0001
MHC I KO A20 p<0.0001
e
aCD47
aCD24
aCD20
aLILRB1
aCD40
aB2M
aCD79b
aCD71
aCD147
aCD38
aCXCR4
aCD98
aCD22
aCD27
PBS
Normalized Fluorescent Area (mm2/well)
Macrophages
Raji p<0.0001
Toledo p<0.0001
SUD-HL-8 p<0.0001

Supplementary Fig. 2 | Comparison of antigens and antibodies evaluated from libraries in the murine and human systems. a, Scatter plot depicting the relationship between binding of 92 shared targets in both human (Raji) and murine systems (A20). The highlighted antigens are the selected antibody hits that exceeded the 95th percentile of functional activity from the co-culture assays with macrophages and B-cell lymphomas as single agents. **b,c,** Scatter plot depicting relationship between the binding of antibody hits and the functional anti-tumor effects across the three different conditions: antibodies alone (in black), in combination with anti-CD47 (in pink), and in combination with anti-CD20 (in blue), in the murine (**b**) and the human system (**c**). **d,e** Heatmaps illustrating the functional efficacy of each antibody hit identified in the screening assay using the WT A20 and MHC I KO A20 murine lymphoma cell lines cultured with or without murine macrophages (**d**) and human lymphoma cell lines (Raji, Toledo, and SUD-HL-8) cultured with or without human macrophages (**e**). Both heatmaps display the normalized fluorescent area (mm^2/well) at the final time point (144 h) of the co-culture. Each data set is normalized to control samples (PBS) for the respective condition. Color intensity represents antibody efficacy, ranging from black (high efficacy; reduced fluorescence indicating fewer tumor cells) to yellow (low efficacy; increased fluorescence indicating more tumor cells). Statistical significance (p-values) was calculated using two-way ANOVA adjusted for multiple comparisons by Sidak method, comparing within each cell line proliferation in the presence or absence of macrophages (**d,** n = 12 treatment conditions with 3 co-cultures per condition; **e,** n = 15 treatment conditions with 2 co-cultures per condition). **a-c,** Data derive from screens presented in Fig. 1. Data represent mean of 2 individual co-cultures for function and one sample for binding for each antibody. Source data are provided as a Source Data file.

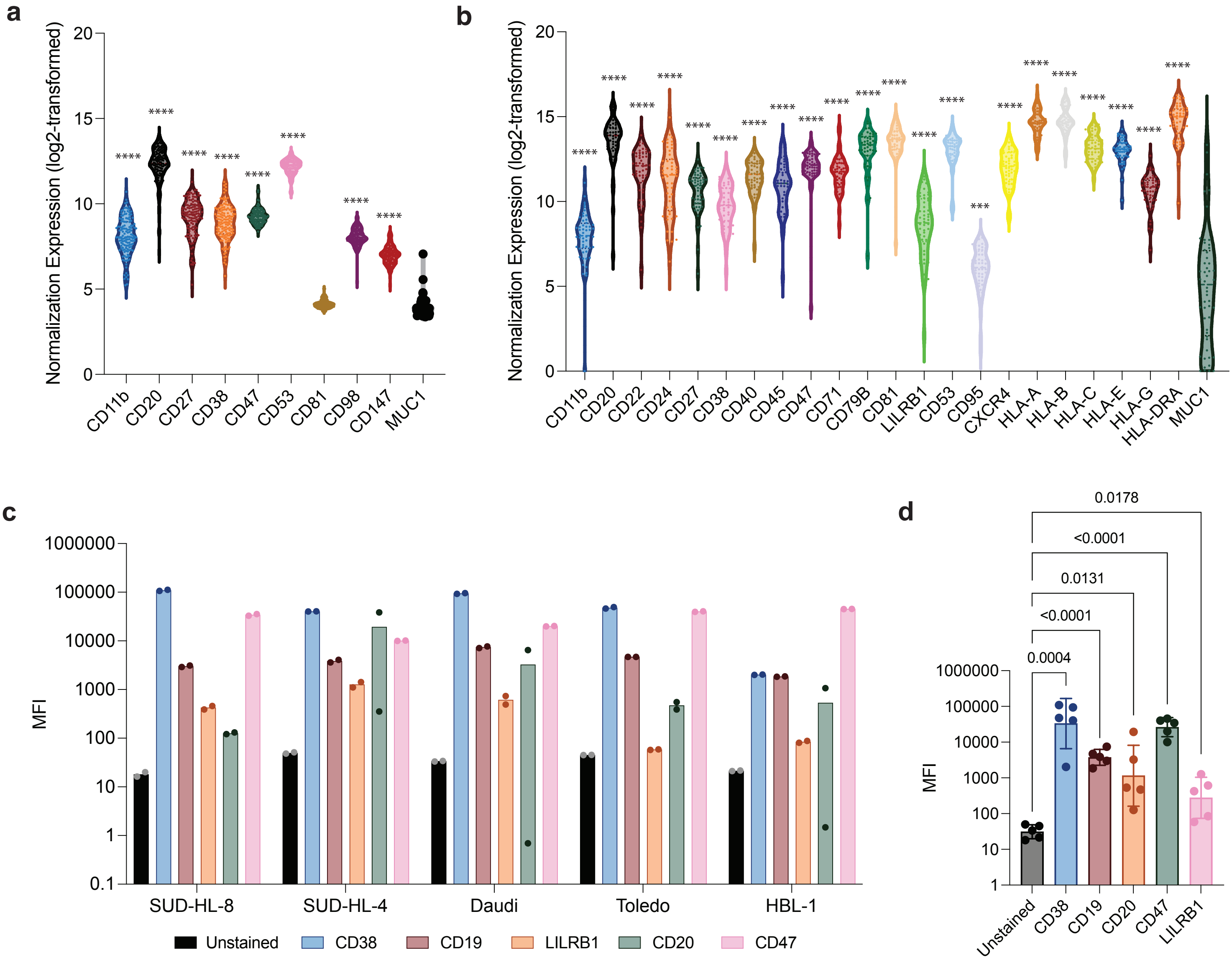
a
Normalization Expression (log2-transformed)
20
15
10
5
0
****
CD11b
CD20
CD27
CD38
CD47
CD53
CD81
CD98
CD147
MUC1
b
Normalization Expression (log2-transformed)
***
CD11b
CD20
CD22
CD24
CD27
CD38
CD40
CD45
CD47
CD71
CD79B
CD81
LILRB1
CD53
CD95
CXCR4
HLA-A
HLA-B
HLA-C
HLA-E
HLA-G
HLA-DRA
MUC1
c
MFI
1000000
100000
10000
1000
100
10
1
0.1
SUD-HL-8
SUD-HL-4
Daudi
Toledo
HBL-1
Unstained
CD38
CD19
LILRB1
CD20
CD47
d
0.0178
<0.0001
0.0131
<0.0001
0.0004
MFI
Unstained
CD38
CD19
CD20
CD47
LILRB1

Supplementary Fig. 3 | The target hits identified in the mouse and human unbiased antibody screens are expressed across different lymphoma patient specimens and cell lines. a,b, Log2-normalized RNA expression of the indicated hits in two lymphoma datasets comprising a total of n = 189 patients (**a**, Chapuy et al.[1], n = 137; **b**, Donati et al.[2] n = 52). Two-way ANOVA with Dunnet test for multiple comparisons were performed to assess differences between each marker expression and control (MUC1). **c,** Flow cytometry binding assays were conducted on five lymphoma cell lines to assess expression of the indicated hits on the cell surface. Points represent two technical replicates from one experiment. **d,** Meta-analysis of means from **c** evaluating surface expression of hits across all cell lines (n = 5 cell lines), with statistical differences in expression among groups compared to control determined by Brown-Forsythe and Welch ANOVA. Source data and exact p values are provided as a Source Data file.

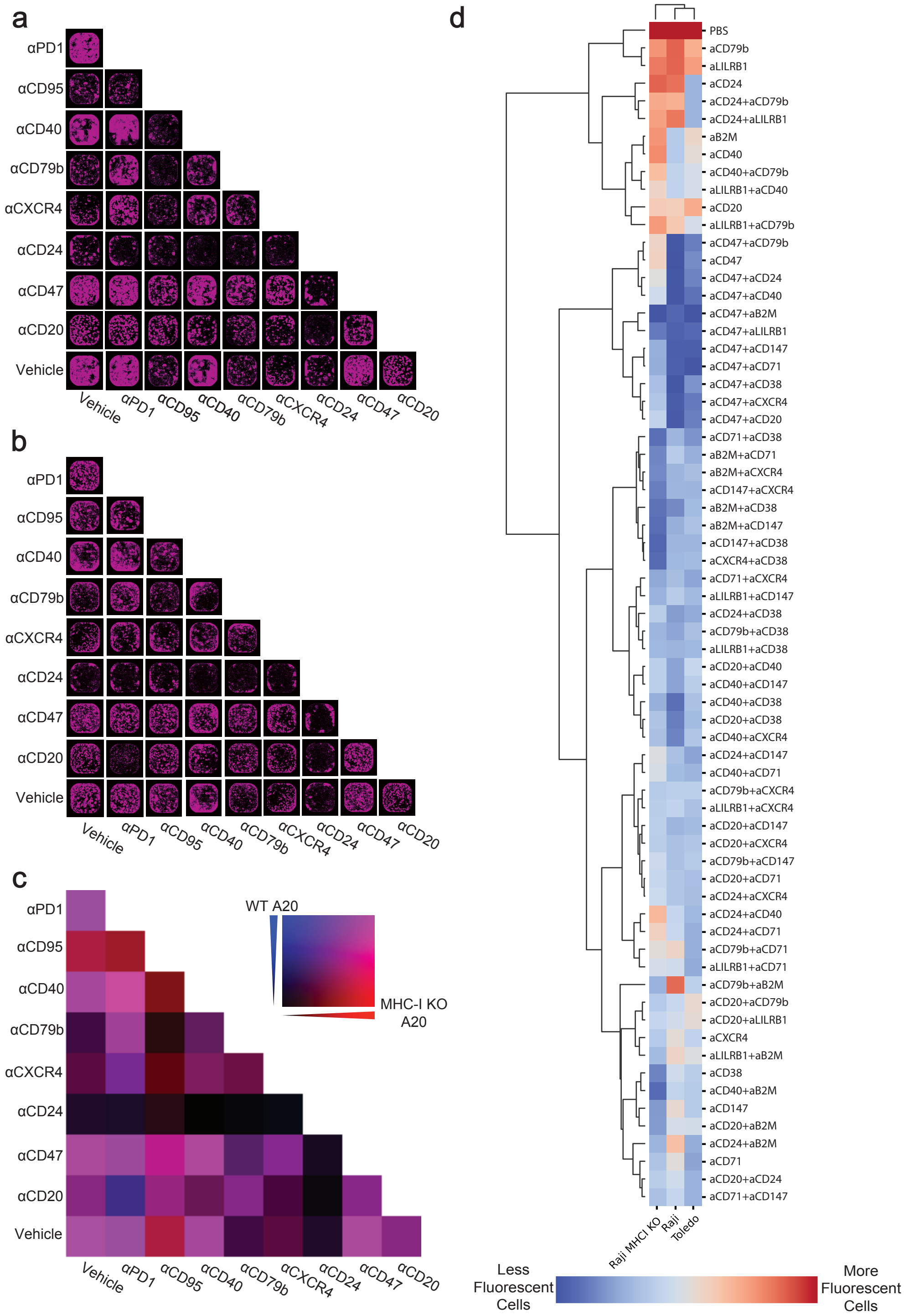

a
αPD1
αCD95
αCD40
αCD79b
αCXCR4
αCD24
αCD47
αCD20
Vehicle
b
c
WT A20
MHC-I KO A20
d
PBS
aCD79b
aLILRB1
aCD24
aCD24+aCD79b
aCD24+aLILRB1
aB2M
aCD40
aCD40+aCD79b
aLILRB1+aCD40
aCD20
aLILRB1+aCD79b
aCD47+aCD79b
aCD47
aCD47+aCD24
aCD47+aCD40
aCD47+aB2M
aCD47+aLILRB1
aCD47+aCD147
aCD47+aCD71
aCD47+aCD38
aCD47+aCXCR4
aCD47+aCD20
aCD71+aCD38
aB2M+aCD71
aB2M+aCXCR4
aCD147+aCXCR4
aB2M+aCD38
aB2M+aCD147
aCD147+aCD38
aCXCR4+aCD38
aCD71+aCXCR4
aLILRB1+aCD147
aCD24+aCD38
aCD79b+aCD38
aLILRB1+aCD38
aCD20+aCD40
aCD40+aCD147
aCD40+aCD38
aCD20+aCD38
aCD40+aCXCR4
aCD24+aCD147
aCD40+aCD71
aCD79b+aCXCR4
aLILRB1+aCXCR4
aCD20+aCD147
aCD20+aCXCR4
aCD79b+aCD147
aCD20+aCD71
aCD24+aCXCR4
aCD24+aCD40
aCD24+aCD71
aCD79b+aCD71
aLILRB1+aCD71
aCD79b+aB2M
aCD20+aCD79b
aCD20+aLILRB1
aCXCR4
aLILRB1+aB2M
aCD38
aCD40+aB2M
aCD147
aCD20+aB2M
aCD24+aB2M
aCD71
aCD20+aCD24
aCD71+aCD147
Raji MHCI KO
Raji
Toledo
Less Fluorescent Cells
More Fluorescent Cells

Supplementary Fig. 4 | Identification of antibody combinations that elicit maximal macrophage-mediated cytotoxicity of B-cell lymphoma. a,b, Murine macrophages were co-cultured with StayGold+ A20 (WT) or MHC-I KO cells. Seven different antibodies identified from our screen and anti-PD1 were tested alone or in combination as indicated. Fluorescent area was measured over time as a representation of growth or elimination of lymphoma cells. Representative images at the last time point (156 h) are shown for each antibody combination for StayGold+ A20 WT (**a**) and StayGold+ A20 MHC-I KO cells (**b**). Images depict whole-well imaging of 384-well plates with well diameter of 2.82 mm for scale. Images are representative of 2 individual co-cultures per antibody combination. **c,** Heat map representing StayGold+ area at last time point (152 h) for WT (blue) and MHC-I-negative (red) A20 cells. Merged data is shown in purple. For both cell lines, lighter color indicates greater StayGold+ area while darker color indicates lesser StayGold+ area. Data represents the mean of 2 individual co-cultures per antibody combination. **d,** Antibody combination studies were performed using human macrophages and GFP+ Raji (WT), GFP+ Raji MHC-I KO, and mScarlet+ Toledo cells treated with 11 different antibodies alone or in combination as indicated. Heatmap depicts the mean normalized fluorescent area of the last time point (152 h) from 2 individual co-culture samples for each antibody or 12 individual co-culture samples for control. Unbiased hierarchical clustering was used to identify antibodies, combinations and cell lines with similar anti-lymphoma properties when co-cultured with macrophages. Source data are provided as a Source Data file.

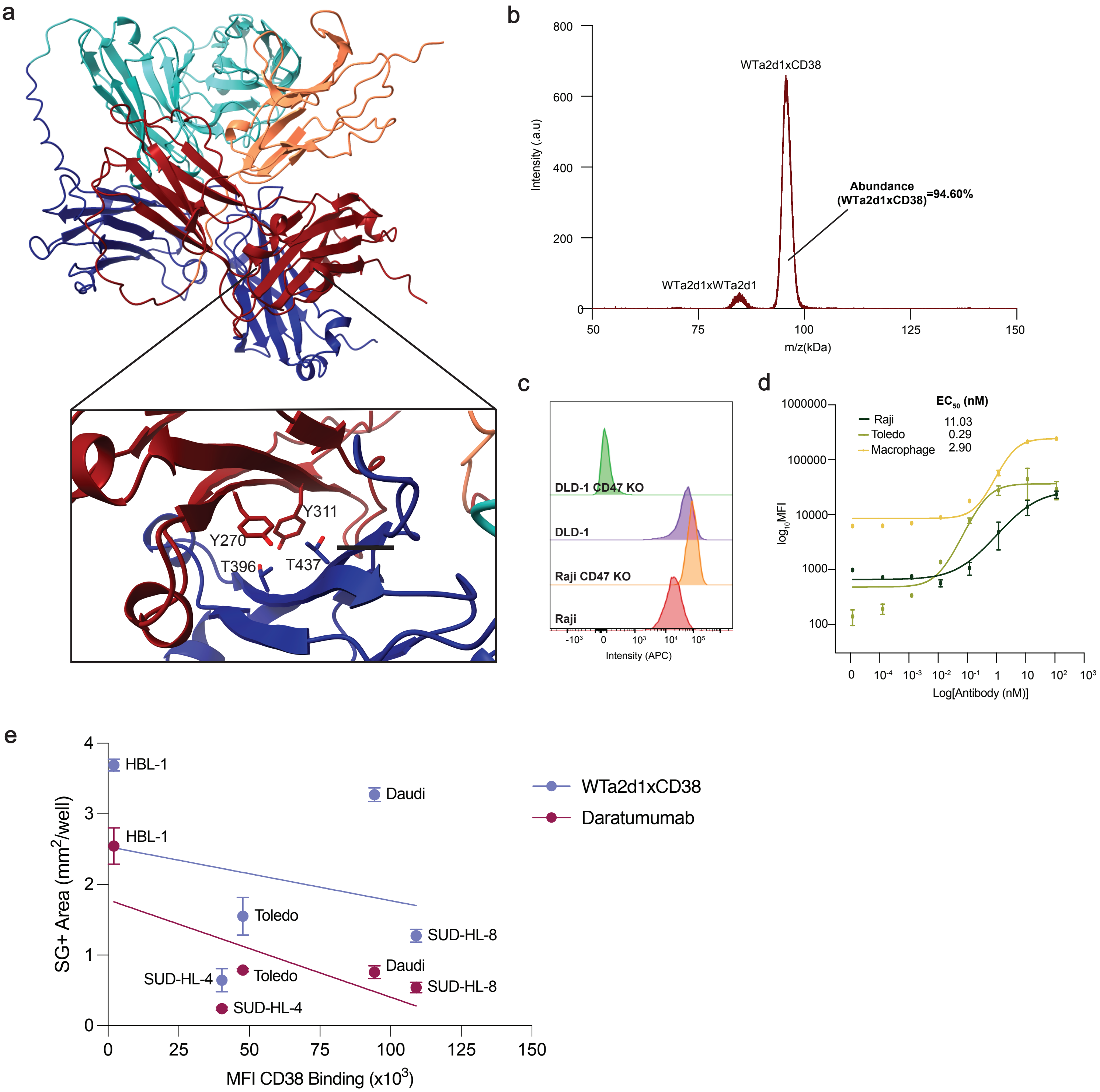
a
Y311
Y270
T396
T437
b
WTa2d1xCD38
Abundance
(WTa2d1xCD38)=94.60%
WTa2d1xWTa2d1
Intensity (a.u)
800
600
400
200
0
50
75
100
125
150
m/z(kDa)
c
DLD-1 CD47 KO
DLD-1
Raji CD47 KO
Raji
-10³
0
10³
10⁴
10⁵
Intensity (APC)
d
EC50 (nM)
Raji 11.03
Toledo 0.29
Macrophage 2.90
1000000
100000
10000
1000
100
log10MFI
0
10⁻⁴
10⁻³
10⁻²
10⁻¹
1
10
10²
10³
Log[Antibody (nM)]
e
HBL-1
Daudi
HBL-1
Toledo
SUD-HL-8
SUD-HL-4
Toledo
Daudi
SUD-HL-8
SUD-HL-4
SG+ Area (mm²/well)
4
3
2
1
0
0
25
50
75
100
125
150
MFI CD38 Binding (x10³)
WTa2d1xCD38
Daratumumab

Supplementary Fig. 5 | Validation of the structure and biophysical characteristics of the WTa2d1xCD38 antibody. a, AlphaFold2 predicted structure of WTa2d1xCD38, a bsAb targeting CD47 and CD38. The Fc region of the anti-CD38 arm is highlighted in blue with its respective scFv region in teal, whereas the Fc region of the WTa2d1 arm is highlighted in red with its respective binding domain in orange. The mutations, "knob" (T270Y, T311Y) and "hole" (Y396T, Y437T) are highlighted at the bottom. **b,** Maldi-TOF analysis of the protein population of the purified bsAb. Based on the theoretical mass of the possible homo and heterodimers, the two main peaks were assigned to WTa2d1xWTa2d1 and WTa2d1xCD38 species. The abundance of WTa2d1xCD38 was calculated as 94.60% based on the peak area (a.u., arbitrary units). Data are representative of $n = 2$ biological replicates, with this sample selected for display based on greater signal intensity observed across the spectrum. (**c**) Histogram showing binding of the bsAb to wild-type versus CD47 knockout (KO) cell lines highlights the ability of the two antibody arms to bind to their respective antigens. Binding was detected with an APC-conjugated anti-human IgG secondary antibody. DLD-1 is a colorectal cancer cell line that expresses CD47 but not CD38. Data represent one binding experiment. **d,** Binding of WTa2d1xCD38 to the indicated human lymphoma cell lines and macrophages combined from 3 donors indicates the cell-based affinity for the antibody. Data represent mean ± SD from 3 technical replicates for all data points except two titration values for Toledo which were excluded due to technical error. **e,** Correlation between expression of CD38 across human lymphoma cell lines and the growth inhibitory effect (SG+ area) in response to WTa2d1xCD38 or daratumumab (10 μg/mL). Regression lines indicate the linear relationship between the functional efficacy and CD38 expression in each condition (WTa2d1xCD38: r=-0.6585, p value=0.2269; daratumumab: r=-0.2495, p=0.6856). Each point represents mean from 3 individual co-cultures presented in Fig. 4b, and CD38 binding data derives from Supplementary Fig. 3c. Source data are provided as a Source Data file.

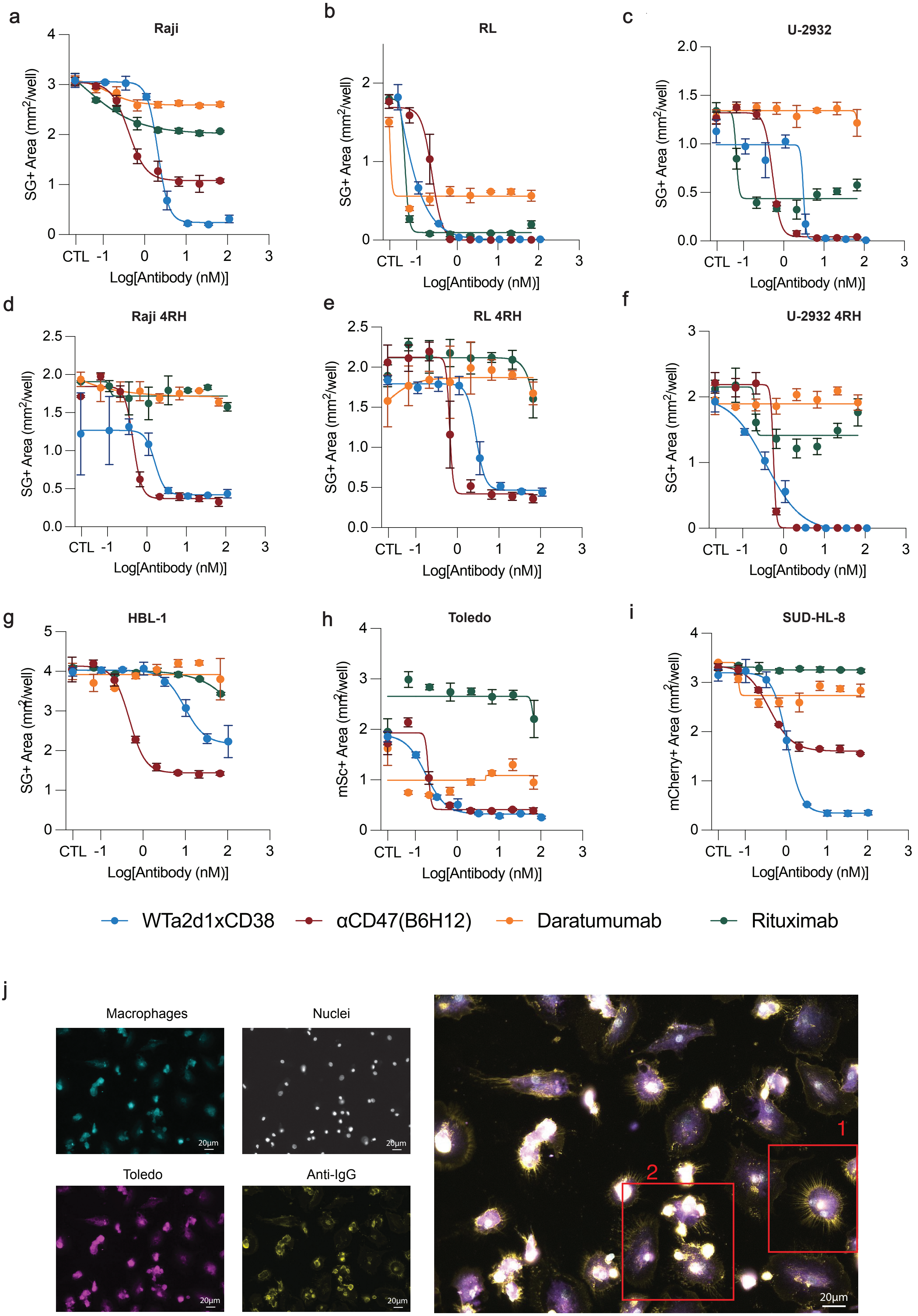
a
Raji
SG+ Area (mm^2/well)
Log[Antibody (nM)]
b
RL
SG+ Area (mm^2/well)
Log[Antibody (nM)]
c
U-2932
SG+ Area (mm^2/well)
Log[Antibody (nM)]
d
Raji 4RH
SG+ Area (mm^2/well)
Log[Antibody (nM)]
e
RL 4RH
SG+ Area (mm^2/well)
Log[Antibody (nM)]
f
U-2932 4RH
SG+ Area (mm^2/well)
Log[Antibody (nM)]
g
HBL-1
SG+ Area (mm^2/well)
Log[Antibody (nM)]
h
Toledo
mSc+ Area (mm^2/well)
Log[Antibody (nM)]
i
SUD-HL-8
mCherry+ Area (mm^2/well)
Log[Antibody (nM)]
CTL -1 0 1 2 3
WTa2d1xCD38
αCD47(B6H12)
Daratumumab
Rituximab
j
Macrophages
Nuclei
Toledo
Anti-IgG
20µm
1
2

Supplementary Fig. 6 | WTa2d1xCD38 bridges the interface between macrophages and cancer cells and is effective across a diversity of lymphoma cell lines, including those that are resistant to rituximab. Human macrophages were co-cultured in 384-well plates with StayGold+ Raji (**a**), RL (**b**), U-2932 (**c**), their respective rituximab-resistant derivatives (**d,** Raji 4RH; **e,** RL 4RH; **f,** U-2932 4RH), and other common lymphoma cell lines (**g,** HBL-1; **h,** Toledo; **i,** SUD-HL-8). **a-i,** Each point depicts the mean fluorescent area at the last time point (144 h) of n = 3 individual co-cultures containing different concentrations of the WTa2d1xCD38, daratumumab, rituximab, or anti-CD47 (clone B6H12). Response curves were fitted using a 4-parameter sigmoidal non-linear regression model. The data at the highest concentration for each antibody is the same as Fig. 4b, with lowest titration for WTa2d1xCD38 used as control. Curves from rituximab-resistant lines also presented in Fig. 4d. **j,** Immunofluorescence images of co-cultures assays with CellTracker green-labeled macrophages, mScarlet+ Toledo cells and 1 µg/mL of WTa2d1xCD38. Cells were fixed with 4% PFA in PBS after 30 minutes incubation with the bsAb at 37°C and 5% CO_2, permeabilized with 0.5% Tween-20 and stained with Alexa-647 anti-human IgG Fcɣ chain specific. The images were visualized using a 40x objective lens with 4-channel fluorescence microscope. Representative of n = 2 independent experiments performed with similar conditions. The figure depicts the images separated by channel, the final merged figure, and the selected regions highlighted in Fig. 4e. Source data are provided as a Source Data file.

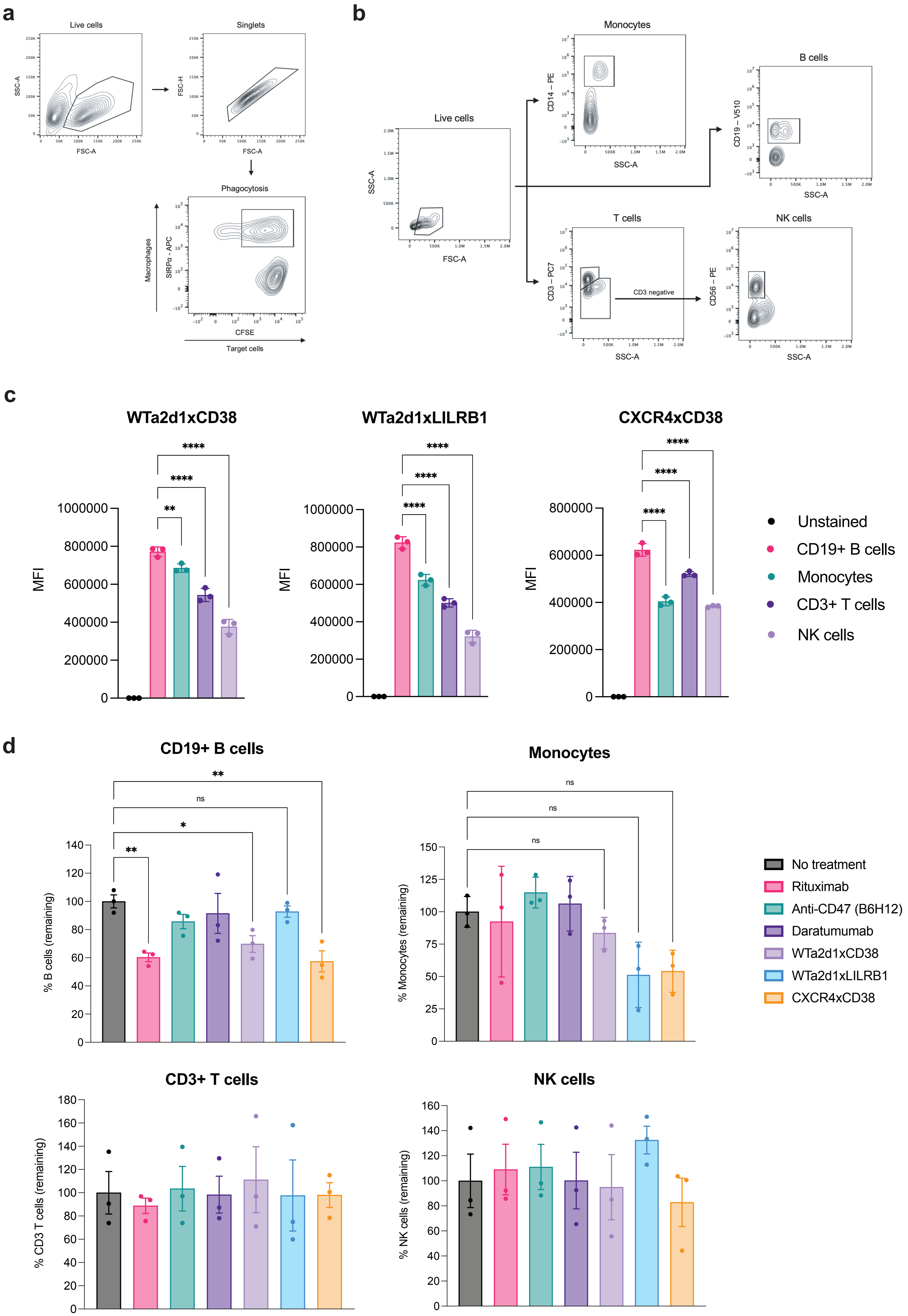
a
Live cells
SSC-A
FSC-A
Singlets
FSC-H
FSC-A
Phagocytosis
Macrophages
SIRPα - APC
CFSE
Target cells
b
Live cells
SSC-A
FSC-A
Monocytes
CD14 – PE
SSC-A
B cells
CD19 – V510
SSC-A
T cells
CD3 – PC7
SSC-A
CD3 negative
NK cells
CD56 – PE
SSC-A
c
WTa2d1xCD38
WTa2d1xLILRB1
CXCR4xCD38
MFI
****
**
Unstained
CD19+ B cells
Monocytes
CD3+ T cells
NK cells
d
CD19+ B cells
% B cells (remaining)
**
ns
*
Monocytes
% Monocytes (remaining)
ns
No treatment
Rituximab
Anti-CD47 (B6H12)
Daratumumab
WTa2d1xCD38
WTa2d1xLILRB1
CXCR4xCD38
CD3+ T cells
% CD3 T cells (remaining)
NK cells
% NK cells (remaining)

Supplementary Fig. 7 | Ex vivo assays to evaluate the effects of three selected bispecific antibodies on immune cell subsets in peripheral blood. a, Example flow cytometry gating strategy for phagocytosis assays using human macrophages (identified by a PE-conjugated anti-SIRPα antibody in this example) and CFSE-labelled target cells. Phagocytosis was measured as the percentage of CFSE+ macrophages. **b,** Example flow cytometry gating strategy to identify immune cell subsets when analyzing PBMCs specimens. **c,** Flow cytometry-based binding assay of WTa2d1xCD38, WTa2d1xLILRB1 and CXCR4xCD38 bsAbs to immune subsets isolated from peripheral blood obtained from healthy donors (n=3). Mean fluorescence intensity (MFI) was assessed. Data depict mean ± SEM. **d,** Percentage of remaining immune cells (B cells, monocytes, T cells, and NK cells) after 24 h treatment with rituximab, anti-CD47 (B6H12), daratumumab, and the indicated bsAbs at 10 µg/mL. Data are normalized to the no treatment control group, and bars represent the mean ± SEM (n=3 peripheral blood donors). (**c-d**) Group differences were analyzed by one-way ANOVA, followed by multiple comparisons versus the non-treated group using the Holm-Sidak correction; statistical significance for indicated comparisons is shown. No significant depletion was observed for T cells or NK cells. ns, not significant, *p<0.05, **p<0.01, ***p<0.001, ****p<0.0001. Source data and exact p values are provided as a Source Data file.

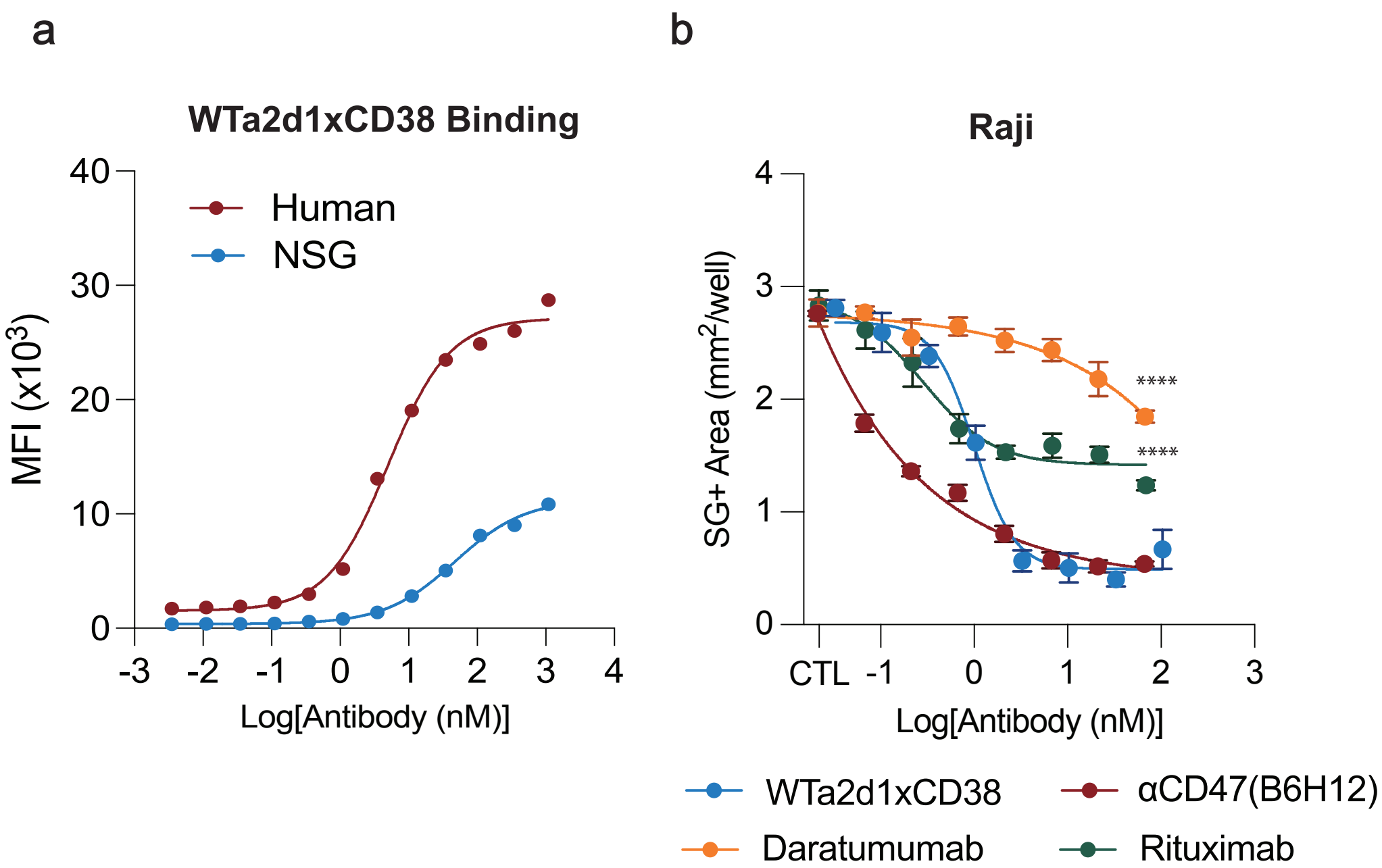
a
WTa2d1xCD38 Binding
Human
NSG
MFI (x10^3)
40
30
20
10
0
-3
-2
-1
0
1
2
3
4
Log[Antibody (nM)]
b
Raji
SG+ Area (mm^2/well)
4
3
2
1
0
CTL
-1
0
1
2
3
Log[Antibody (nM)]
****
****
WTa2d1xCD38
αCD47(B6H12)
Daratumumab
Rituximab

Supplementary Fig. 8 | Macrophage-dependent variations in antibody responses in the mouse and human systems. a, Flow cytometry-based binding assay of the WTa2d1xCD38 bsAb in bone-marrow-derived mouse macrophages and monocyte-derived human macrophages. Data represent mean from three technical replicates. **b,** Cancer cell area remaining after 144-hour co-culture of human lymphoma cell lines with mouse-derived macrophages in the presence of various antibodies at their maximum concentration (10 µg/mL). Antibodies tested include WTa2d1xCD38, anti-CD47 (clone B6H12), rituximab, and daratumumab**.** Growth inhibitory concentration (IC_{50}) curves showing the potency of WTa2d1xCD38, anti-CD47 (clone B6H12), rituximab, and daratumumab antibodies (10 µg/mL) in co-culture of NSG-derived mouse macrophages and Raji cell line from n = 3 individual co-culture specimens. Two-way ANOVA with Tukey adjustment for multiple comparisons was used to evaluate the difference between groups. ****p<0.0001. Source data and exact p values are provided as a Source Data file.

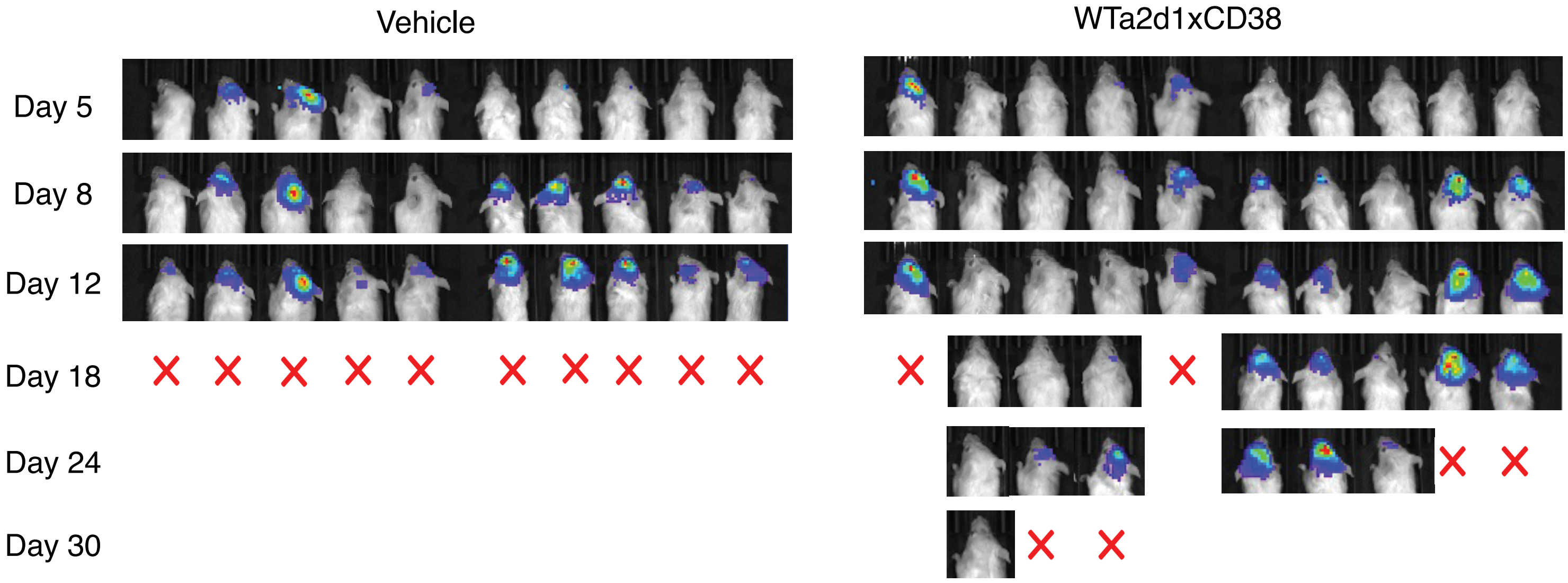

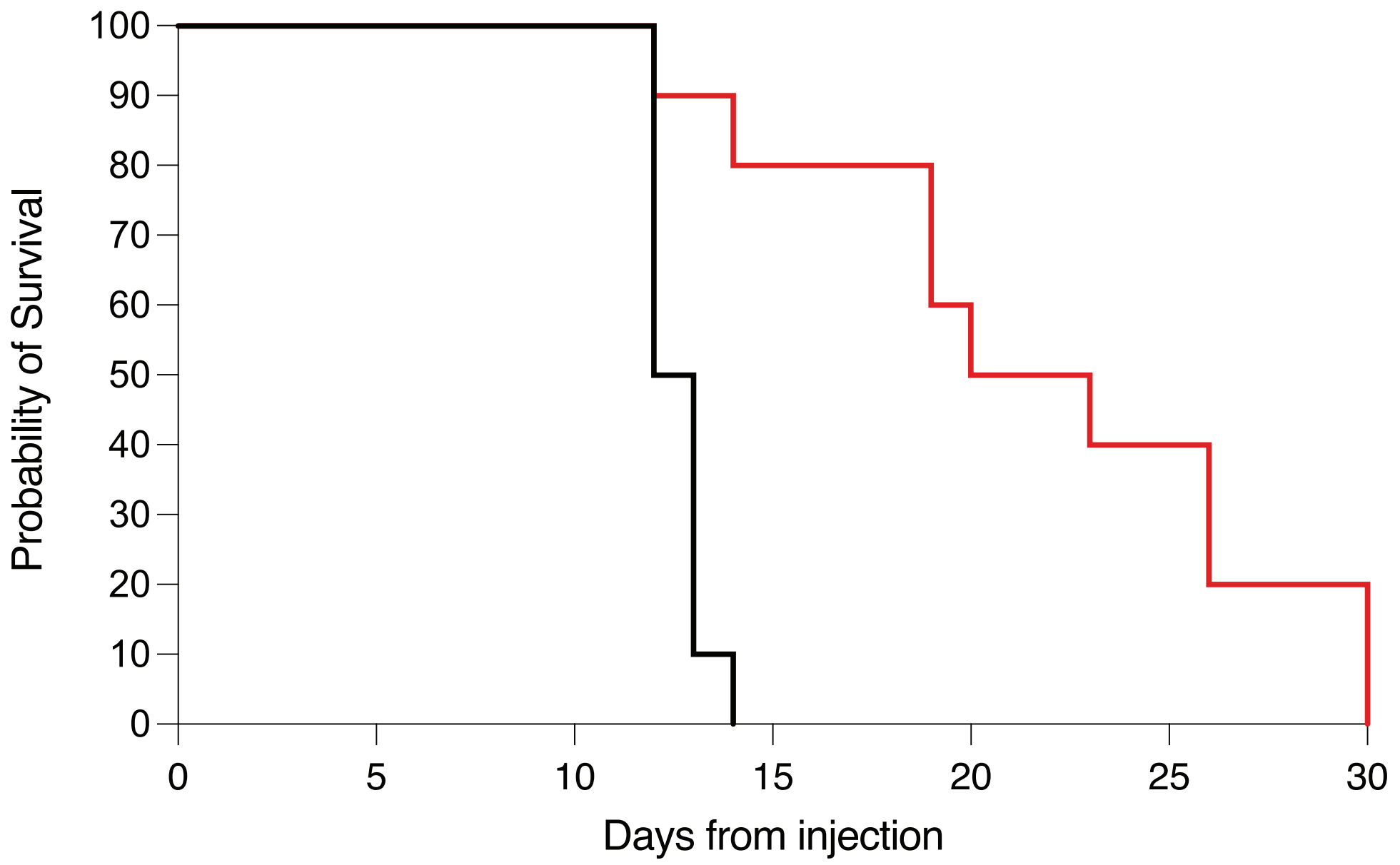

Median survival	
PBS	WTa2d1xCD38
12.5	21.5

p=0.0001

Supplementary Fig. 9 | Meta-analysis of survival from in vivo experiments using WTa2d1xCD38 in a mouse model of primary CNS lymphoma. Bioluminescence imaging using luciferase reporters of CNS tumors was employed throughout the experiment period, starting at day 5 post-tumor injection. Survival curves compare the efficacy of WTa2d1xCD38 treatment (red) versus vehicle control (black). Data were pooled from two independent experiments, including experiment presented in Fig. 5f, that were conducted identically (n = 5 mice per group per experiment; total n = 10 per group). Mice were treated with vehicle control or 200 µg WTa2d1xCD38 three times per week. Log-rank Mantel-Cox test was used to evaluate the difference in survival between vehicle and WTa2d1xCD38 treatment conditions. Source data are provided as a Source Data file.

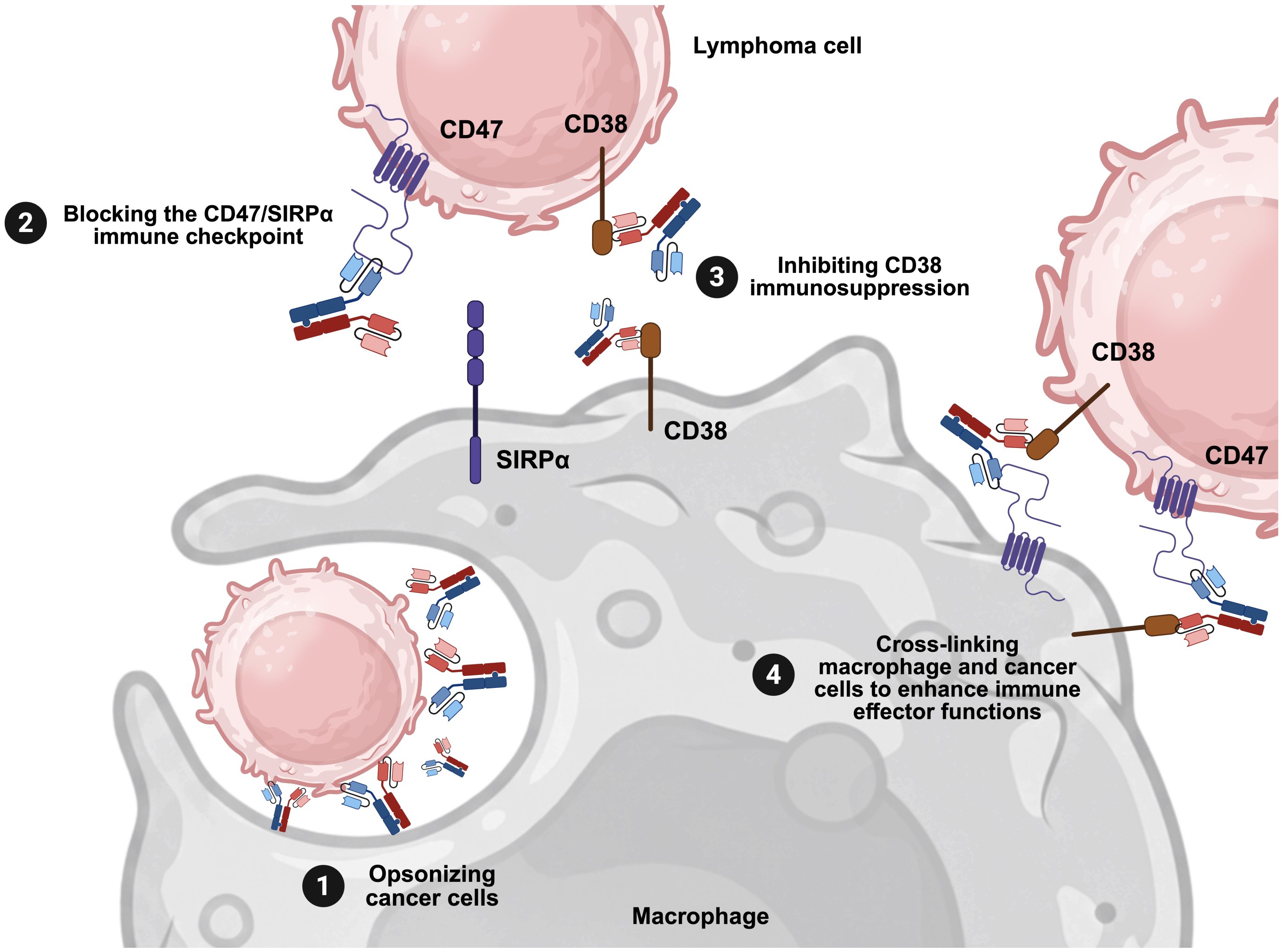

Lymphoma cell
CD47
CD38
2 Blocking the CD47/SIRPα immune checkpoint
3 Inhibiting CD38 immunosuppression
SIRPα
CD38
CD38
CD47
4 Cross-linking macrophage and cancer cells to enhance immune effector functions
1 Opsonizing cancer cells
Macrophage

Supplementary Fig. 10 | Putative mechanisms of action for the WTa2d1xCD38 bispecific antibody. Representation of the biological functions performed by the bsAb to enhance macrophage-mediated destruction of B-cell lymphoma cells. The WTa2d1xCD38 bispecific can act as an opsonin and engage Fc receptors on macrophages (1). It can also block immunosuppressive pathways on both the cancer and macrophage cell surface (2 and 3). Additionally, it can enhance the biophysical interactions of macrophages and B-cell lymphoma cells, bringing their cell membranes in close proximity to promote phagocytosis (4). Together, these functions maximally activate macrophage-mediated cytotoxicity of aggressive B-cell lymphoma cells. Created in BioRender. Pages-Geli, C. (2026) https://BioRender.com/4xmfpff.

Supplementary Table 1 | A list of antibodies used in this study.

Product	Catalog nº	Reactivity	Target	Clone	Specific Catalog nº	Supplier	Concentration used	Dilution Factor
		Mouse	CD2	RM2-5	533109	BD	6.55 µg/ml	
		Mouse	CD4	GK1.5	553727	BD	6.55 µg/ml	
		Mouse	CD5	53-7.3	553017	BD	6.55 µg/ml	
		Mouse	CD8A	53-6.7	558733	BD	6.55 µg/ml	
		Mouse	CD9	KMC8	553758	BD	6.55 µg/ml	
		Mouse	CD11a	M17/4	550528	BD	6.55 µg/ml	
		Mouse	CD11b	M1/70	553308	BD	6.55 µg/ml	
		Mouse	CD13	R3-242		BD	6.55 µg/ml	
		Mouse	CD14	RMC5-3	553738	BD	6.55 µg/ml	
		Mouse	CD16/CD32	2.4G2	553141	BD	6.55 µg/ml	
		Mouse	CD18	C71/16		BD	6.55 µg/ml	
		Mouse	CD19	1D3	553783	BD	6.55 µg/ml	
		Mouse	CD21/CD35	7G6	553817	BD	6.55 µg/ml	
		Mouse	CD23	B3B4		BD	6.55 µg/ml	
		Mouse	CD24	M1/69	557436	BD	6.55 µg/ml	
		Mouse	CD25	PC61		BD	6.55 µg/ml	
		Mouse	CD26	H194-112		BD	6.55 µg/ml	
		Mouse	CD29	9EG7	553715	BD	6.55 µg/ml	
		Mouse	CD31	MEC13.3		BD	6.55 µg/ml	
		Mouse	CD34	RAM34	553731	BD	6.55 µg/ml	
		Mouse	CD35	8C12	558768	BD	6.55 µg/ml	
		Mouse	CD38	HIT2	555458	BD	6.55 µg/ml	
		Mouse	CD41	MWREG30	553847	BD	6.55 µg/ml	
		Mouse	CD43	S7	552366	BD	6.55 µg/ml	
		Mouse	CD44	IM7	550538	BD	6.55 µg/ml	
		Mouse	CD45	30-F11	550539	BD	6.55 µg/ml	
		Mouse	CD45R	RA3-6B2	553084	BD	6.55 µg/ml	
		Mouse	CD45RA	14.8		BD	6.55 µg/ml	
		Mouse	CD45RC	DNL-1.9		BD	6.55 µg/ml	
		Mouse	CD47	MIAP301	555297	BD	6.55 µg/ml	
		Mouse	CD49b	DX5	553855	BD	6.55 µg/ml	
		Mouse	CD49d	9C10 (MRF4.B)	553314	BD	6.55 µg/ml	
		Mouse	CD49e	5H10-27	553319	BD	6.55 µg/ml	
		Mouse	CD51	RMV-7		BD	6.55 µg/ml	
		Mouse	CD53	OX-79		BD	6.55 µg/ml	
		Mouse	CD62E	10E9.6	553749	BD	6.55 µg/ml	
		Mouse	CD62L	MEL-14	553148	BD	6.55 µg/ml	
		Mouse	CD70	FR70		BD	6.55 µg/ml	
		Mouse	CD71	C2	553264	BD	6.55 µg/ml	
		Mouse	CD72 b/c	JY/93		BD	6.55 µg/ml	
		Mouse	CD73	TY/23	550738	BD	6.55 µg/ml	
		Mouse	CD80	1G10/B7		BD	6.55 µg/ml	
		Mouse	CD83	MICHEL-19		BD	6.55 µg/ml	
		Mouse	CD86	PO3		BD	6.55 µg/ml	
		Mouse	CD90.2	30-H12	553009	BD	6.55 µg/ml	
		Mouse	CD94	18D3		BD	6.55 µg/ml	
		Mouse	CD98	H202-141		BD	6.55 µg/ml	
		Mouse	CD102	3C4 (MIC2/4)	553326	BD	6.55 µg/ml	
		Mouse	CD103	M290	553699	BD	6.55 µg/ml	
		Mouse	CD104	346-11A	553745	BD	6.55 µg/ml	
		Mouse	CD106	429 (MVCAM.A)	550547	BD	6.55 µg/ml	
		Mouse	CD117	2B8	553352	BD	6.55 µg/ml	
		Mouse	CD121a	35F5	553693	BD	6.55 µg/ml	
		Mouse	CD121b	4E2		BD	6.55 µg/ml	
		Mouse	CD122	TM-BETA1		BD	6.55 µg/ml	
		Mouse	CD123	5B11		BD	6.55 µg/ml	
		Mouse	CD124	MIL4R-M1	551853	BD	6.55 µg/ml	
		Mouse	CD125	T21		BD	6.55 µg/ml	
		Mouse	CD126	D7715A7		BD	6.55 µg/ml	
		Mouse	CD127	B12-1		BD	6.55 µg/ml	
		Mouse	CD131	JORO50		BD	6.55 µg/ml	
		Mouse	CD132	TUGM2		BD	6.55 µg/ml	
		Mouse	CD134	OX-86		BD	6.55 µg/ml	

Mouse Cell Surface Marker Screening Panel	562208	Mouse	CD135	A2F10.1	569553	BD	6.55 µg/ml	
		Mouse	CD137	1AH2		BD	6.55 µg/ml	
		Mouse	CD138	281-2	553712	BD	6.55 µg/ml	
		Mouse	CD140a	APA5	558774	BD	6.55 µg/ml	
		Mouse	CD144	11D4.1	555289	BD	6.55 µg/ml	
		Mouse	CD147	RL73		BD	6.55 µg/ml	
		Mouse	CD153	RM153		BD	6.55 µg/ml	
		Mouse	CD162	2PH1		BD	6.55 µg/ml	
		Mouse	CD172a	P84	552371	BD	6.55 µg/ml	
		Mouse	CD179a	R3/VPREB		BD	6.55 µg/ml	
		Mouse	CD179b	LM34		BD	6.55 µg/ml	
		Mouse	CD180	RP/14	552128	BD	6.55 µg/ml	
		Mouse	CD184 (CXCR4)	12G5	555972	BD	6.55 µg/ml	
		Mouse	CD185 (CXCR5)	2G8	551961	BD	6.55 µg/ml	
		Mouse	CD195	C34-3448	559921	BD	6.55 µg/ml	
		Mouse	CD197	4B12/CCR7.1		BD	6.55 µg/ml	
		Mouse	CD200	OX-90		BD	6.55 µg/ml	
		Mouse	CD209a	5H10/CIRE		BD	6.55 µg/ml	
		Mouse	CD210	1B1.3A		BD	6.55 µg/ml	
		Mouse	CD223	C9B7W	552379	BD	6.55 µg/ml	
		Mouse	CD244.1	C9.1		BD	6.55 µg/ml	
		Mouse	CD252	RM134L		BD	6.55 µg/ml	
		Mouse	CD253	N2B2		BD	6.55 µg/ml	
		Mouse	CD254 (RANKL)	IK22-5		BD	6.55 µg/ml	
		Mouse	CD267	8F10		BD	6.55 µg/ml	
		Mouse	CD273	TY25	757927	BD	6.55 µg/ml	
		Mouse	CD274	MIH5	567839	BD	6.55 µg/ml	
		Mouse	CD278	7E.17G9		BD	6.55 µg/ml	
		Mouse	CD284	MTS510		BD	6.55 µg/ml	
		Mouse	CD309	Avas 12a1		BD	6.55 µg/ml	
		Mouse	CD314	CX5		BD	6.55 µg/ml	
		Mouse	CD326	G8.8	552370	BD	6.55 µg/ml	
		Mouse	CD335 (NKp46)	29A1.4	560754	BD	6.55 µg/ml	
		Mouse	4-1BB Ligand	TKS-1		BD	6.55 µg/ml	
		Mouse	Crry/p65	1F2		BD	6.55 µg/ml	
		Mouse	Dendritic Cells	33D1		BD	6.55 µg/ml	
		Mouse	Early B Lineage	493		BD	6.55 µg/ml	
		Mouse	F4/80-like Receptor	6F12	552958	BD	6.55 µg/ml	
		Mouse	GITR	DTA-1	558213	BD	6.55 µg/ml	
		Mouse	I-A/I-E	2G9	553621	BD	6.55 µg/ml	
		Mouse	IL-21 Receptor	4A9		BD	6.55 µg/ml	
		Mouse	Integrin β7 chain	FIB27		BD	6.55 µg/ml	
		Mouse	LPAM-1	DATK32		BD	6.55 µg/ml	
		Mouse	Ly-6A/E	E13-161.7	553333	BD	6.55 µg/ml	
		Mouse	Ly-6D	49-H4	557360	BD	6.55 µg/ml	
		Mouse	Ly-6G	1A8	551459	BD	6.55 µg/ml	
		Mouse	Ly-6G/Ly-6C	RB6-8C5	550291	BD	6.55 µg/ml	
		Mouse	CD107b	M3/84	553322	BD	6.55 µg/ml	
		Mouse	MAdCAM-1	MECA-89		BD	6.55 µg/ml	
		Mouse	MD-1	MD14		BD	6.55 µg/ml	
		Mouse	NKG2A/C/E	20D5	550518	BD	6.55 µg/ml	
		Mouse	NKT/NK Cell Antigen	U5A2-13		BD	6.55 µg/ml	
		Mouse	Panendothelial Cell Ag	MECA-32	550563	BD	6.55 µg/ml	
		Mouse	PIR-A/B	6C1		BD	6.55 µg/ml	
		Mouse	Pre-BCR	SL156		BD	6.55 µg/ml	
		Mouse	Siglec-F	E50-2440	552125	BD	6.55 µg/ml	
		Mouse	Syndecan-4	KY/8.2	550350	BD	6.55 µg/ml	
		Mouse	T/B Cell Activation Ag	GL7		BD	6.55 µg/ml	
		Mouse	Erythroid Cells	TER-119	550565	BD	6.55 µg/ml	

Mouse	Igλ1,λ2,λ3 Light Chain	R26-46	553432	BD	6.55 µg/ml	
Mouse	IgD	11-26C.2A	553438	BD	6.55 µg/ml	
Mouse	IgE	R35-72	553413	BD	6.55 µg/ml	
Mouse	IgM	R6-60.2	553405	BD	6.55 µg/ml	
Mouse	ACD28	37.51	553295	BD	6.55 µg/ml	
Mouse	KLRG1	2F1	562190	BD	6.55 µg/ml	
Mouse	Vγ 3 TCR	536		BD	6.55 µg/ml	
Mouse	CD3e	145-2C11	567115	BD	6.55 µg/ml	
Mouse	CD11c	HL3	553799	BD	6.55 µg/ml	
Mouse	CD27	LG.3A10		BD	6.55 µg/ml	
Mouse	CD309	MCD30.1		BD	6.55 µg/ml	
Mouse	CD40	HM40-3	553721	BD	6.55 µg/ml	
Mouse	CD42d	1C2		BD	6.55 µg/ml	
Mouse	CD48	HM48-1		BD	6.55 µg/ml	
Mouse	CD54	3E2	553250	BD	6.55 µg/ml	
Mouse	CD55	RIKO-5		BD	6.55 µg/ml	
Mouse	CD61 (Integrin β3)	2C9.G2	550541	BD	6.55 µg/ml	
Mouse	CD69	H1.2F3		BD	6.55 µg/ml	
Mouse	CD79b	HM79B	555302	BD	6.55 µg/ml	
Mouse	CD81	EAT2		BD	6.55 µg/ml	
Mouse	CD95 (Fas)	JO2	554255	BD	6.55 µg/ml	
Mouse	CD119 (IFNgR1)	2E2		BD	6.55 µg/ml	
Mouse	CD120a	55R-286		BD	6.55 µg/ml	
Mouse	CD120b	TR75-89	559916	BD	6.55 µg/ml	
Mouse	CD152	UC10-4F10	553719	BD	6.55 µg/ml	
Mouse	CD154	MR1	569050	BD	6.55 µg/ml	
Mouse	CD279	J43	551891	BD	6.55 µg/ml	
Mouse	γδ-TCR	GL3	553175	BD	6.55 µg/ml	
Mouse	gp49 Receptor	H1.1		BD	6.55 µg/ml	
Mouse	H2-M3	130	553893	BD	6.55 µg/ml	
Mouse	IFN-γR β Chain	MOB-47		BD	6.55 µg/ml	
Mouse	TCR β Chain	H57-597	553166	BD	6.55 µg/ml	
Mouse	CD22.2	CY34.1		BD	6.55 µg/ml	
Mouse	CD45.1	A20		BD	6.55 µg/ml	
Mouse	CD45.2	104	552023	BD	6.55 µg/ml	
Mouse	CD64 a/b AlloAgs	X54-5/7.1		BD	6.55 µg/ml	
Mouse	CD72 a/b/d AlloAgs	K10.6		BD	6.55 µg/ml	
Mouse	CD157	BP-3		BD	6.55 µg/ml	
Mouse	CD212	114	556999	BD	6.55 µg/ml	
Mouse	CD244.2	2B4	557451	BD	6.55 µg/ml	
Mouse	$H\text{-}2D^b$	KH95	554213	BD	6.55 µg/ml	
Mouse	$H\text{-}2K^b$	AF6-88.5	553567	BD	6.55 µg/ml	
Mouse	$H\text{-}2K^d$	SF1-1.1	553563	BD	6.55 µg/ml	
Mouse	$H\text{-}2K^K$	AF3-12.1		BD	6.55 µg/ml	
Mouse	$H\text{-}2K^Q$	KH114		BD	6.55 µg/ml	
Mouse	$H\text{-}2K^S$	KH49		BD	6.55 µg/ml	
Mouse	IFN-α/β receptor 1	MAR1-5A3	561183	BD	6.55 µg/ml	
Mouse	LY-49C LY-49I	5E6	553273	BD	6.55 µg/ml	
Mouse	LY-51	BP-1	551853	BD	6.55 µg/ml	
Mouse	NK-1.1	PK136	553162	BD	6.55 µg/ml	
Mouse	Pre-TCR a chain	2F5	552407	BD	6.55 µg/ml	
Mouse	QA-1B	6A8.6F10.1A6	559827	BD	6.55 µg/ml	
Mouse	SSEA-1	MC480	560079	BD	6.55 µg/ml	
Mouse	SSEA-4	MC813-70	560073	BD	6.55 µg/ml	
Rat	IgG1,κ IC	R3-34	553922	BD	6.55 µg/ml	
Rat	IgG1,λ IC	A110-1	559157	BD	6.55 µg/ml	
Rat	IgG2a,κ IC	R35-95	559073	BD	6.55 µg/ml	
Rat	IgG2a,λ IC	B39-4	553996	BD	6.55 µg/ml	
Rat	IgG2b,κ IC	A95-1	553986	BD	6.55 µg/ml	
Rat	IgG2c,κ IC	A23-1	553982	BD	6.55 µg/ml	
Rat	IgM,κ IC	R4-22	553940	BD	6.55 µg/ml	

		Hamster	IgG1,κ IC	A19-3	550344	BD	6.55 µg/ml	
		Hamster	IgG1,λ IC	G235-2356	553951	BD	6.55 µg/ml	
		Hamster	IgG2,κ IC	B81-3	559277	BD	6.55 µg/ml	
		Hamster	IgG2,λ IC	Ha4/8	553961	BD	6.55 µg/ml	
		Hamster	IgG3,λ IC	A19-4		BD	6.55 µg/ml	
		Hamster	IgM,λ IC	G235-1	553957	BD	6.55 µg/ml	
		Mouse	IgG1,κ IC	MOPC-31C	557273	BD	6.55 µg/ml	
		Mouse	IgG2a,κ IC	G155-178	554645	BD	6.55 µg/ml	
		Mouse	IgG2b,κ IC	MPC-11	557351	BD	6.55 µg/ml	
		Mouse	IgG3,κ IC	A112-3	553486	BD	6.55 µg/ml	
		Mouse	IgGM,κ IC	G155-228	553472	BD	6.55 µg/ml	
		Human	CD1a	HI149	555805	BD	6.55 µg/ml	
		Human	CD1b	M-T101		BD	6.55 µg/ml	
		Human	CD1d	CD1d42	550254	BD	6.55 µg/ml	
		Human	CD2	RPA-2.10	555323	BD	6.55 µg/ml	
		Human	CD3	HIT3a	550367	BD	6.55 µg/ml	
		Human	CD4	RPA-T4	555344	BD	6.55 µg/ml	
		Human	CD4v4	L120		BD	6.55 µg/ml	
		Human	CD5	L17F12		BD	6.55 µg/ml	
		Human	CD6	M-T605	555356	BD	6.55 µg/ml	
		Human	CD7	M-T701	555359	BD	6.55 µg/ml	
		Human	CD8a	SK1		BD	6.55 µg/ml	
		Human	CD8b	2ST8.5H7		BD	6.55 µg/ml	
		Human	CD9	M-L13	555370	BD	6.55 µg/ml	
		Human	CD10	HI10a	555373	BD	6.55 µg/ml	
		Human	CD11a	G43-25B	555378	BD	6.55 µg/ml	
		Human	CD11b	D12		BD	6.55 µg/ml	
		Human	CD11c	B-ly6	555390	BD	6.55 µg/ml	
		Human	CD13	WM15	555393	BD	6.55 µg/ml	
		Human	CD14	M5E2	555395	BD	6.55 µg/ml	
		Human	CD15	HI98	555400	BD	6.55 µg/ml	
		Human	CD15s	CSLEX1	551344	BD	6.55 µg/ml	
		Human	CD16	3G8	555403	BD	6.55 µg/ml	
		Human	CD18	6.7	555922	BD	6.55 µg/ml	
		Human	CD19	HIB19	555409	BD	6.55 µg/ml	
		Human	CD20	2H7	555621	BD	6.55 µg/ml	
		Human	CD21	B-ly4	555421	BD	6.55 µg/ml	
		Human	CD22	HIB22	555423	BD	6.55 µg/ml	
		Human	CD23	EBVCS-5		BD	6.55 µg/ml	
		Human	CD24	ML5	555426	BD	6.55 µg/ml	
		Human	CD25	M-A251	555430	BD	6.55 µg/ml	
		Human	CD26	M-A261	555435	BD	6.55 µg/ml	
		Human	CD27	M-T271	555439	BD	6.55 µg/ml	
		Human	CD28	L293	340975	BD	6.55 µg/ml	
		Human	CD29	HUTS-21	556048	BD	6.55 µg/ml	
		Human	CD30	BerH8	555827	BD	6.55 µg/ml	
		Human	CD31	WM59	550389	BD	6.55 µg/ml	
		Human	CD32	FLI8.26	555447	BD	6.55 µg/ml	
		Human	CD33	HIM3-4	555625	BD	6.55 µg/ml	
		Human	CD34	581	550390	BD	6.55 µg/ml	
		Human	CD35	E11	555451	BD	6.55 µg/ml	
		Human	CD36	CB38 (NL07)	555453	BD	6.55 µg/ml	
		Human	CD37	M-B371	555456	BD	6.55 µg/ml	
		Human	CD38	HIT2	555458	BD	6.55 µg/ml	
		Human	CD39	TU66		BD	6.55 µg/ml	
		Human	CD40	5C3	555587	BD	6.55 µg/ml	
		Human	CD41a	HIP8	555465	BD	6.55 µg/ml	
		Human	CD41b	HIP2	555468	BD	6.55 µg/ml	
		Human	CD42a	ALMA.16	558816	BD	6.55 µg/ml	
		Human	CD42b	HIP1	562255	BD	6.55 µg/ml	
		Human	CD43	1G10	555474	BD	6.55 µg/ml	
		Human	CD44	G44-26	555476	BD	6.55 µg/ml	
		Human	CD45	HI30	555480	BD	6.55 µg/ml	
		Human	CD45RA	HI100	555486	BD	6.55 µg/ml	
		Human	CD45RB	MT4		BD	6.55 µg/ml	
		Human	CD45RO	UCHL1	555491	BD	6.55 µg/ml	

ning Panel

Human	CD46	E4.3	555948	BD	6.55 µg/ml	
Human	CD47	B6H12	556043	BD	6.55 µg/ml	
Human	CD48	TU145	555758	BD	6.55 µg/ml	
Human	CD49a	SR84	559594	BD	6.55 µg/ml	
Human	CD49b	AK-7	555497	BD	6.55 µg/ml	
Human	CD49c	C3 II.1		BD	6.55 µg/ml	
Human	CD49d	9F10	555501	BD	6.55 µg/ml	
Human	CD49e	VC5	555651	BD	6.55 µg/ml	
Human	CD50	TU41		BD	6.55 µg/ml	
Human	CD51/61	23C6	555504	BD	6.55 µg/ml	
Human	CD53	HI29		BD	6.55 µg/ml	
Human	CD54	LB-2	559047	BD	6.55 µg/ml	
Human	CD55	IA10	555691	BD	6.55 µg/ml	
Human	CD56	B159	555514	BD	6.55 µg/ml	
Human	CD57	NK-1	555618	BD	6.55 µg/ml	
Human	CD58	1C3	555919	BD	6.55 µg/ml	
Human	CD59	p282(H19)	555761	BD	6.55 µg/ml	
Human	CD61	VI-PL2	555752	BD	6.55 µg/ml	
Human	CD62E	68-5H11	555648	BD	6.55 µg/ml	
Human	CD62L	Dreg 56	555541	BD	6.55 µg/ml	
Human	CD62P	AK-4	555522	BD	6.55 µg/ml	
Human	CD63	H5C6	556019	BD	6.55 µg/ml	
Human	CD64	10.1	555525	BD	6.55 µg/ml	
Human	CD66 (a,c,d,e)	B1.1/CD66	551354	BD	6.55 µg/ml	
Human	CD66b	G10F5	555723	BD	6.55 µg/ml	
Human	CD66f	IID10		BD	6.55 µg/ml	
Human	CD69	FN50	555529	BD	6.55 µg/ml	
Human	CD70	Ki-24	555833	BD	6.55 µg/ml	
Human	CD71	M-A712	555534	BD	6.55 µg/ml	
Human	CD72	J4-117	555917	BD	6.55 µg/ml	
Human	CD73	AD2	550256	BD	6.55 µg/ml	
Human	CD74	MB741	555538	BD	6.55 µg/ml	
Human	CD75	LN1		BD	6.55 µg/ml	
Human	CD77	5B5	551352	BD	6.55 µg/ml	
Human	CD79b	CB3-1	555678	BD	6.55 µg/ml	
Human	CD80	L307.4	557223	BD	6.55 µg/ml	
Human	CD81	JS-81	555675	BD	6.55 µg/ml	
Human	CD83	HB15e	556854	BD	6.55 µg/ml	
Human	CD84	2G7		BD	6.55 µg/ml	
Human	CD85	GHI/75	555941	BD	6.55 µg/ml	
Human	CD86	2331 (FUN-1)	555655	BD	6.55 µg/ml	
Human	CD87	VIM5	555767	BD	6.55 µg/ml	
Human	CD88	D53-1473	550493	BD	6.55 µg/ml	
Human	CD89	A59	555685	BD	6.55 µg/ml	
Human	CD90	5E10	555593	BD	6.55 µg/ml	
Human	CD91	A2MR-alpha 2	550495	BD	6.55 µg/ml	
Human	CDw93	R139	552954	BD	6.55 µg/ml	
Human	CD94	HP-3D9	555887	BD	6.55 µg/ml	
Human	CD95	DX2	555671	BD	6.55 µg/ml	
Human	CD97	VIM3b		BD	6.55 µg/ml	
Human	CD98	UM7F8	556074	BD	6.55 µg/ml	
Human	CD99	TU12	555687	BD	6.55 µg/ml	
Human	CD99R	HIT4		BD	6.55 µg/ml	
Human	CD100	A8		BD	6.55 µg/ml	
Human	CD102	CBR-1C2/2		BD	6.55 µg/ml	
Human	CD103	Ber-ACT8	550258	BD	6.55 µg/ml	
Human	CD105	266	555690	BD	6.55 µg/ml	
Human	CD106	51-10C9	555645	BD	6.55 µg/ml	
Human	CD107a	H4A3	555798	BD	6.55 µg/ml	
Human	CD107b	H4B4	555803	BD	6.55 µg/ml	
Human	CD108	KS-2		BD	6.55 µg/ml	
Human	CD109	TEA 2/16	556039	BD	6.55 µg/ml	
Human	CD112	R2.525	551056	BD	6.55 µg/ml	
Human	CD114	LMM741		BD	6.55 µg/ml	
Human	CD116	M5D12		BD	6.55 µg/ml	
Human	CD117	YB5.B8	555713	BD	6.55 µg/ml	

BD Lyoplate™ Human Cell Surface Marker Scree	560747	Human	CD118	12D3		BD	6.55 µg/ml	
		Human	CD119	GIR-208	557531	BD	6.55 µg/ml	
		Human	CD120a	MABTNFR1-A1	552535	BD	6.55 µg/ml	
		Human	CD121a	HIL1R-M1		BD	6.55 µg/ml	
		Human	CD121b	MNC2	552401	BD	6.55 µg/ml	
		Human	CD122	Mik-beta 3		BD	6.55 µg/ml	
		Human	CD123	9F5	555642	BD	6.55 µg/ml	
		Human	CD124	hIL4R-M57	551894	BD	6.55 µg/ml	
		Human	CD126	M5	551462	BD	6.55 µg/ml	
		Human	CD127	hIL-7R-M21	552853	BD	6.55 µg/ml	
		Human	CD128b	6C6	555932	BD	6.55 µg/ml	
		Human	CD130	AM64	555756	BD	6.55 µg/ml	
		Human	CD134	ACT35	555836	BD	6.55 µg/ml	
		Human	CD135	4G8	558995	BD	6.55 µg/ml	
		Human	CD137	4B4-1	555955	BD	6.55 µg/ml	
		Human	CD137 Ligand	C65-485	559445	BD	6.55 µg/ml	
		Human	CD138	Mi15	551902	BD	6.55 µg/ml	
		Human	CD140a	αR1	556001	BD	6.55 µg/ml	
		Human	CD140b	28D4	558820	BD	6.55 µg/ml	
		Human	CD141	1A4	559780	BD	6.55 µg/ml	
		Human	CD142	HTF-1	550252	BD	6.55 µg/ml	
		Human	CD144	55-7H1	555661	BD	6.55 µg/ml	
		Human	CD146	P1H12	550314	BD	6.55 µg/ml	
		Human	CD147	HIM6	555961	BD	6.55 µg/ml	
		Human	CD150	A12		BD	6.55 µg/ml	
		Human	CD151	14A2.H1	556056	BD	6.55 µg/ml	
		Human	CD152	BNI3	550405	BD	6.55 µg/ml	
		Human	CD153	D2-1173	550052	BD	6.55 µg/ml	
		Human	CD154	TRAP1	555698	BD	6.55 µg/ml	
		Human	CD158a	HP-3E4	556061	BD	6.55 µg/ml	
		Human	CD158b	CH-L	559783	BD	6.55 µg/ml	
		Human	CD161	DX12	556079	BD	6.55 µg/ml	
		Human	CD162	KPL-1	556052	BD	6.55 µg/ml	
		Human	CD163	GHI/61	556017	BD	6.55 µg/ml	
		Human	CD164	N6B6	551296	BD	6.55 µg/ml	
		Human	CD165	SN2		BD	6.55 µg/ml	
		Human	CD166	3A6	559260	BD	6.55 µg/ml	
		Human	CD171	5G3	554273	BD	6.55 µg/ml	
		Human	CD172b	B4B6		BD	6.55 µg/ml	
		Human	CD177	MEM-166	551899	BD	6.55 µg/ml	
		Human	CD178	NOK-1	556372	BD	6.55 µg/ml	
		Human	CD180	G28-8	551890	BD	6.55 µg/ml	
		Human	CD181	5A12	555937	BD	6.55 µg/ml	
		Human	CD183	1C6/CXCR3	557183	BD	6.55 µg/ml	
		Human	CD184	12G5	555972	BD	6.55 µg/ml	
		Human	CD193	5E8		BD	6.55 µg/ml	
		Human	CD195	2D7/CCR5	555991	BD	6.55 µg/ml	
		Human	CD196	11A9	559560	BD	6.55 µg/ml	
		Human	CD197	2H4	550937	BD	6.55 µg/ml	
		Human	CD200	MRC OX-104	552023	BD	6.55 µg/ml	
		Human	CD205	MG38	558531	BD	6.55 µg/ml	
		Human	CD206	19.2	555953	BD	6.55 µg/ml	
		Human	CD209	DCN46	551186	BD	6.55 µg/ml	
		Human	CD220	3B6/IR	559954	BD	6.55 µg/ml	
		Human	CD221	3B7		BD	6.55 µg/ml	
		Human	CD226	DX11	559787	BD	6.55 µg/ml	
		Human	CD227	HMPV	555925	BD	6.55 µg/ml	
		Human	CD229	HLy9.1.25		BD	6.55 µg/ml	
		Human	CD231	M3-3D9 (SN1a)		BD	6.55 µg/ml	
		Human	CD234a	GA-R2 (HIR2)	555569	BD	6.55 µg/ml	
		Human	CD243	17F9	557001	BD	6.55 µg/ml	
		Human	CD244	2-69	550814	BD	6.55 µg/ml	
		Human	CD255	CARL-1		BD	6.55 µg/ml	
		Human	CD268	11C1		BD	6.55 µg/ml	
		Human	CD271	CD40-1457		BD	6.55 µg/ml	
		Human	CD273	MIH18	562178	BD	6.55 µg/ml	

		Human	CD274	MIH1	565959	BD	6.55 µg/ml	
		Human	CD275	2D3/B7-H2	552501	BD	6.55 µg/ml	
		Human	CD278	DX29	557801	BD	6.55 µg/ml	
		Human	CD279	MIH4	557823	BD	6.55 µg/ml	
		Human	CD282	11G7		BD	6.55 µg/ml	
		Human	CD305	DX26	550810	BD	6.55 µg/ml	
		Human	CD309	89106		BD	6.55 µg/ml	
		Human	CD314	1D11	552866	BD	6.55 µg/ml	
		Human	CD321	M.AB.F11		BD	6.55 µg/ml	
		Human	CDw327	E20-1232		BD	6.55 µg/ml	
		Human	CDw328	F023-420	552885	BD	6.55 µg/ml	
		Human	CD329	E10-286		BD	6.55 µg/ml	
		Human	CD335	9E2/NKp46	557911	BD	6.55 µg/ml	
		Human	CD336	P44-8		BD	6.55 µg/ml	
		Human	CD337	P30-15		BD	6.55 µg/ml	
		Human	CD338	5D3	551226	BD	6.55 µg/ml	
		Human	CD304	Neu24.7		BD	6.55 µg/ml	
		Human	αβTCR	T10B9.1A-31	555546	BD	6.55 µg/ml	
		Human	β2-microglobulin	TU99	555550	BD	6.55 µg/ml	
		Human	BLTR-1	203/14F11		BD	6.55 µg/ml	
		Human	CLIP	CerCLIP		BD	6.55 µg/ml	
		Human	CMRF-44	CMRF44		BD	6.55 µg/ml	
		Human	CMRF-56	CMRF56		BD	6.55 µg/ml	
		Human	EGF Receptor	EGFR1	555996	BD	6.55 µg/ml	
		Human	fMLP receptor	5F1		BD	6.55 µg/ml	
		Human	γδTCR	B1	555715	BD	6.55 µg/ml	
		Human	HPC	BB9		BD	6.55 µg/ml	
		Human	HLA-A,B,C	G46-2.6	555551	BD	6.55 µg/ml	
		Human	HLA-A2	BB7.2	551230	BD	6.55 µg/ml	
		Human	HLA-DQ	TU169	555562	BD	6.55 µg/ml	
		Human	HLA-DR	G46-6 (L243)	555809	BD	6.55 µg/ml	
		Human	HLA-DR, DP, DQ	TU39	555557	BD	6.55 µg/ml	
		Human	Invariant NK T	6B11		BD	6.55 µg/ml	
		Human	Disialoganglioside GD2	14.62a		BD	6.55 µg/ml	
		Human	MIC A/B	6D4		BD	6.55 µg/ml	
		Human	NKB1	DX9	555964	BD	6.55 µg/ml	
		Human	SSEA-1	MC480	560079	BD	6.55 µg/ml	
		Human	SSEA-4	MC813-70	560073	BD	6.55 µg/ml	
		Human	TRA-1-60	TRA-1-60	560071	BD	6.55 µg/ml	
		Human	TRA-1-81	TRA-1-81	560072	BD	6.55 µg/ml	
		Human	Vβ 23	AHUT7		BD	6.55 µg/ml	
		Human	Vβ 8	JR2		BD	6.55 µg/ml	
		Human	CD326	EBA-1		BD	6.55 µg/ml	
		Human	Ms IgM IC	G155-228	555581	BD	6.55 µg/ml	
		Human	CD49f	GoH3	555734	BD	6.55 µg/ml	
		Human	CD104	439-9B	555719	BD	6.55 µg/ml	
		Human	CD120b	hTNFR-M1	551311	BD	6.55 µg/ml	
		Human	CD132	TuGh4	555896	BD	6.55 µg/ml	
		Human	CD201	RCR-252		BD	6.55 µg/ml	
		Human	CD210	3F9	556012	BD	6.55 µg/ml	
		Human	CD212	2B6/12beta 2		BD	6.55 µg/ml	
		Human	CD267	1A1-K21-M22		BD	6.55 µg/ml	
		Human	CD294	BM16	558412	BD	6.55 µg/ml	
		Human	SSEA-3	MC631	568844	BD	6.55 µg/ml	
		Human	CLA	HECA-452	555946	BD	6.55 µg/ml	
		Human	Integrin β7	FIB504	555943	BD	6.55 µg/ml	
		Rat	IgM IC	R4-22	553940	BD	6.55 µg/ml	
		Rat	IgG1 IC	R3-34	553922	BD	6.55 µg/ml	
		Rat	IgG2a IC	R35-95	559073	BD	6.55 µg/ml	
		Rat	IgG2b IC	A95-1	553986	BD	6.55 µg/ml	
		Mouse	IgG1 IC	MOP-21		BD	6.55 µg/ml	
		Mouse	IgG2a IC	G155-178	554645	BD	6.55 µg/ml	
		Mouse	IgG2b IC	27-35	555740	BD	6.55 µg/ml	
		Mouse	IgG3 IC	J606	555577	BD	6.55 µg/ml	

InVivoMAb anti-mouse CD20	BE0356	Mouse	CD20	MB20-11	BE0356	BioXcell	10 µg/ml	
InVivoMAb anti-mouse/human/rat CD47 (IAP)	BE0283	Mouse/Human/Rat	CD47	MIAP410	BE0283	BioXcell	10 µg/ml	
InVivoMAb anti-mouse CD24	BE0360	Mouse	CD24	M1/69	BE0360	BioXcell	10 µg/ml	
InVivoMAb anti-mouse PD-1 (CD279)	BE0146	Mouse	PD1	RMP1-14	BE0146	BioXcell	10 µg/ml	
inVivoSIM anti-human CD20 (Rituximab Biosimilar)	SIM0008	Human	CD20	Rituximab	SIM0008	Bioxcell	10 µg/ml	
Rituximab	HY-P9913	Human	CD20	Rituximab	HY-P9913	MedChemExpress	10 µg/ml	
Daratumumab	HY-P9915	Human	CD38	Daratumumab	HY-P9915	MedChemExpress	10 µg/ml	
InVivoMAb anti-human CD47	BE0019-1	Human	CD47	B6H12	BE0019-1	Bioxcell	10 µg/ml	
Biotin Goat Anti-Rat Ig		Rat	Ig	Poly1271	554014	BD	1.25µg/ml	
Biotin Goat Anti-Mouse Ig		Mouse	Ig	Poly1270	550337	BD	1.25µg/ml	
Biotin Goat Anti-Armenian Hamster Ig		Armenian Hasmter	Ig			BD	0.6 µg/mL	
Biotin Goat Anti-Syrian Hamster Ig		Syrian Hamster	Ig			BD	0.6 µg/mL	
Alexa Fluor® 647 Streptavidin		-	Streptavidin			BD	0.5 µg/mL	
AlexaFluor® 647 Goat Anti-Mouse Ig		Mouse	Ig			BD	1.25µg/ml	
AlexaFluor® 647 Goat Anti-Rat Ig		Rat	Ig			BD	1.25µg/ml	
Alexa Fluor® 647 AffiniPure® Goat Anti-Human IgG, Fcγ fragment specific	109-605-008	Human	Ig-Fcy	Polyclonal	109-605-008	Jackson Immunoresearch Laboratories		250
Peroxidase AffiniPure® Goat Anti-Human IgG, Fcγ fragment specific	109-035-190	Human	Ig-Fcy	Polyclonal	109-035-008	Jackson Immunoresearch Laboratories		5000
Goat anti-mouse IgG(H+L) Secondary Antibody, HRP	62-6520	Mouse	Ig-H+L	Polyclonal	62-6520	Invitrogen		5000
Goat anti-Human Kappa Light Chain Secondary Antibody, HRP	A18853	Human	Ig Kappa	Polyclonal	A18853	Invitrogen		5000
Fibronectin Monoclonal Antibody (FN-3), eBioscience™	14-9869-82	Human	Fibronectin	3F12	14-9869-82	Invitrogen		1000
APC anti-human CD172a/b (SIRPα/β) Antibody	323810	Human	Sirpa	SE5A5	323810	Biolegend		100
PE anti-human CD56 Antibody	985902	Human	CD56	QA17A16	985902	Biolegend		100
PE anti-human CD14 Antibody	325606	Human	CD14	HCD14	325606	Biolegend		100
PE/Cyanine7 anti-human CD3 Antibody	317334	Human	CD3	OKT3	317334	Biolegend		100
Brilliant Violet 510™ anti-human CD19 Antibody	302242	Human	CD19	HIB19	302242	Biolegend		100
APC anti-human CD38 Antibody	980308	Human	CD38	HIT2	980308	Biolegend		100
APC anti-human CD19 Antibody	982406	Human	CD19	HIB19	982406	Biolegend		100
APC anti-human CD47 Antibody	323124	Human	CD47	CC2C6	323124	Biolegend		100
APC anti-human CD85j (LILRB1) Antibody	333720	Human	CD85j	ILT2	333720	Biolegend		100

Supplementary References

1. Chapuy, B. *et al.* Molecular subtypes of diffuse large B cell lymphoma are associated with distinct pathogenic mechanisms and outcomes. *Nat. Med.* **24**, 679–690 (2018).
2. Donati, B. *et al.* Gene expression profile unveils diverse biological effect of serum vitamin D in Hodgkin's and diffuse large B-cell lymphoma. *Hematol. Oncol.* **39**, 205–214 (2021).
