# Supplementary material for: High-throughput engineering of bispecific antibodies to enhance macrophage-mediated cytotoxicity of B-cell lymphoma
Source: Nat Commun. 2026 Aug 1;17:8491. doi: 10.1038/s41467-026-76180-5 (PMC13482096; doi:10.1038/s41467-026-76180-5)
Supplement: Supplementary file 2 [file 41467_2026_76180_MOESM2_ESM.pdf]

Corresponding author(s): Kipp Weiskopf, M.D., Ph.D.

Last updated by author(s): Jun 15, 2026

Reporting Summary

Nature Portfolio wishes to improve the reproducibility of the work that we publish. This form provides structure for consistency and transparency in reporting. For further information on Nature Portfolio policies, see our Editorial Policies and the Editorial Policy Checklist.

Statistics

For all statistical analyses, confirm that the following items are present in the figure legend, table legend, main text, or Methods section.

n/a	Confirmed	
☐	☒	The exact sample size (n) for each experimental group/condition, given as a discrete number and unit of measurement
☐	☒	A statement on whether measurements were taken from distinct samples or whether the same sample was measured repeatedly
☐	☒	The statistical test(s) used AND whether they are one- or two-sided<br>*Only common tests should be described solely by name; describe more complex techniques in the Methods section.*
☐	☒	A description of all covariates tested
☐	☒	A description of any assumptions or corrections, such as tests of normality and adjustment for multiple comparisons
☐	☒	A full description of the statistical parameters including central tendency (e.g. means) or other basic estimates (e.g. regression coefficient) AND variation (e.g. standard deviation) or associated estimates of uncertainty (e.g. confidence intervals)
☐	☒	For null hypothesis testing, the test statistic (e.g. F, t, r) with confidence intervals, effect sizes, degrees of freedom and P value noted<br>*Give P values as exact values whenever suitable.*
☒	☐	For Bayesian analysis, information on the choice of priors and Markov chain Monte Carlo settings
☒	☐	For hierarchical and complex designs, identification of the appropriate level for tests and full reporting of outcomes
☐	☒	Estimates of effect sizes (e.g. Cohen's d, Pearson's r), indicating how they were calculated

Our web collection on statistics for biologists contains articles on many of the points above.

Software and code

Policy information about availability of computer code

Data collection	Raw data were acquired using the Incucyte S3 Live-Cell Imaging System; IVIS Spectrum imaging system (Living Image software); Keyence BZ-X810 fluorescence microscope (BZ-X Viewer/BZ-X Analyzer software); BD LSRFortessa flow cytometer (FACSDiva software); Beckman Coulter CytoFLEX flow cytometer (CytExpert software); Azure 200 gel imaging system; and Bruker Microflex LT MALDI-TOF.<br><br>No custom algorithms, code, or software were used for data collection.
Data analysis	Incucyte S3 Analysis Software (version v2018B-2025A); Python (version 3.10.0); GraphPad Prism (9.2.0, 10.2, and 11), Microsoft Excel version 16.109.1, FlowJo (version 10.9), ImageJ (version 1.54p), Living Image (version 4.7.4), Bruker Daltonics flex Analysis version 3.3.80.0.<br><br>No custom algorithms, code, or software was used for data analysis.

For manuscripts utilizing custom algorithms or software that are central to the research but not yet described in published literature, software must be made available to editors and reviewers. We strongly encourage code deposition in a community repository (e.g. GitHub). See the Nature Portfolio guidelines for submitting code & software for further information.

Data

Policy information about availability of data

All manuscripts must include a data availability statement. This statement should provide the following information, where applicable:

- Accession codes, unique identifiers, or web links for publicly available datasets
- A description of any restrictions on data availability
- For clinical datasets or third party data, please ensure that the statement adheres to our policy

The authors declare that the data supporting the findings of this study are available within the paper and its supplementary information files.

Research involving human participants, their data, or biological material

Policy information about studies with human participants or human data. See also policy information about sex, gender (identity/presentation), and sexual orientation and race, ethnicity and racism.

Reporting on sex and gender	Experiments were performed with macrophages derived from de-identified human blood specimens. We expect that both male and female specimens are included, but donor-specific sex and gender information is not available for the specimens used.
Reporting on race, ethnicity, or other socially relevant groupings	Experiments were performed with macrophages derived from de-identified human blood specimens. Demographic information is not available for these specimens.
Population characteristics	Experiments were performed with macrophages derived from de-identified human blood specimens. Demographic information is not available for these specimens.
Recruitment	Not applicable. No Human Subject research was performed in this study.
Ethics oversight	Not applicable. The secondary use of de-identified human biological specimens in this study does not meet criteria for Human Subject research. Our studies were determined to be exempt from review by the MIT Committee on Use of Humans as Experimental Subjects and the Beth Israel Deaconess Medical Center Committee on Clinical Investigations.

Note that full information on the approval of the study protocol must also be provided in the manuscript.

Field-specific reporting

Please select the one below that is the best fit for your research. If you are not sure, read the appropriate sections before making your selection.

[x] Life sciences [ ] Behavioural & social sciences [ ] Ecological, evolutionary & environmental sciences

For a reference copy of the document with all sections, see nature.com/documents/nr-reporting-summary-flat.pdf

Life sciences study design

All studies must disclose on these points even when the disclosure is negative.

Sample size	For screening and binding assays, our study included 173 antibodies in the murine system and 241 antibodies in the human system. For bispecific experiments, we engineered and tested 156 distinct molecular entities. All co-culture experiments were performed using a minimum of two distinct co-cultures for each condition except as indicated otherwise. In vivo experiments were performed with n = 5 mice per experimental cohort.
Data exclusions	In some experiments, a small number of replicate values were excluded due to technical errors (e.g., bubbles in the wells affecting cell growth or fluorescent area measurements). The corresponding entries are noted in the Source Data file next to the relevant antibody or replicate values. These excluded values are not expected to impact the conclusions of our study.
Replication	For co-culture assays, we conducted experiments using a minimum of two distinct co-cultures per condition except as indicated otherwise. In most cases, the demonstration of biological variability was achieved by repeating experiments across multiple different cell lines or samples. For in vivo experiments, we used n = 5 mice per experimental cohort.
Randomization	Mice were randomly assigned to age and sex-matched treatment cohorts.
Blinding	Tumor volume measurements were performed by an investigator who was blinded to experimental hypotheses.

Reporting for specific materials, systems and methods

We require information from authors about some types of materials, experimental systems and methods used in many studies. Here, indicate whether each material, system or method listed is relevant to your study. If you are not sure if a list item applies to your research, read the appropriate section before selecting a response.

Materials & experimental systems

n/a	Involved in the study	
[ ]	[x]	Antibodies
[ ]	[x]	Eukaryotic cell lines
[x]	[ ]	Palaeontology and archaeology
[ ]	[x]	Animals and other organisms
[x]	[ ]	Clinical data
[x]	[ ]	Dual use research of concern
[x]	[ ]	Plants

Methods

n/a	Involved in the study	
[x]	[ ]	ChIP-seq
[ ]	[x]	Flow cytometry
[x]	[ ]	MRI-based neuroimaging

Antibodies

Antibodies used

A list of antibodies used in this study, including supplier, clone name, catalog number and dilution, is provided in Supplementary Table 1 and summarized in the Methods section.

Validation

Manufacturer-provided validation material for key antibodies can be found at:

Mouse Cell Surface Marker Screening Panel: https://www.bdbiosciences.com/content/dam/bdb/products/global/reagents/flow-cytometry-reagents/research-reagents/panels-multicolor-cocktails-ruo/562xxx/5622xx/562208_base/pdf/562208.pdf
Human Cell Surface Marker Screening Panel: https://www.bdbiosciences.com/content/dam/bdb/products/global/reagents/flow-cytometry-reagents/research-reagents/panels-multicolor-cocktails-ruo/560xxx/5607xx/560747_base/pdf/560747.pdf

Anti-mouse CD20 (MB20-11), BioXcell: https://bioxcell.com/pub/media/tds/BP0356-TDS.pdf
Anti-mouse CD47 (MIAP410), BioXcell: https://bioxcell.com/pub/media/tds/BE0283-TDS.pdf
Anti-human CD47 (B6H12), BioXcell: https://bioxcell.com/pub/media/tds/BE0019-1-TDS.pdf
Anti-human CD20 (Rituximab biosimilar), BioXcell: https://bioxcell.com/pub/media/tds/SIM0008-TDS.pdf
CD24 (M1/69), BioXcell: https://bioxcell.com/pub/media/tds/BE0360-tds.pdf
PD1 (RMP1-14), BioXcell: https://bioxcell.com/pub/media/tds/BE0146-TDS.pdf

Eukaryotic cell lines

Policy information about cell lines and Sex and Gender in Research

Cell line source(s)

A20 (ATCC, cat #TIB-208)
Raji (ATCC, cat #CCL-86)
SU-DHL-4 (Koch Institute High throughput Services Core)
Toledo (Koch Institute High throughput Services Core)
SU-DHL-8 (Gift from Catherine Wu's lab - Dana Farber Cancer Institute)
HBL-1 (Gift from Catherine Wu's lab - Dana Farber Cancer Institute)
Daudi (Gift from Catherine Wu's lab - Dana Farber Cancer Institute)
U-2932 (Gift from Francisco J. Hernandez-Ilizaliturri lab - Roswell Park Cancer Institute)
U-2932 4RH (Gift from Francisco J. Hernandez-Ilizaliturri lab - Roswell Park Cancer Institute)
Raji 4RH (Gift from Francisco J. Hernandez-Ilizaliturri lab - Roswell Park Cancer Institute)
RL (Gift from Francisco J. Hernandez-Ilizaliturri lab - Roswell Park Cancer Institute)
RL 4RH (Gift from Francisco J. Hernandez-Ilizaliturri lab - Roswell Park Cancer Institute)

Authentication

Cell lines were obtained from trusted sources and/or authenticated by STR profiling as necessary.

Mycoplasma contamination

Cell lines were tested for Mycoplasma contamination and banked for use in subsequent experiments.

Commonly misidentified lines
(See ICLAC register)

None of the cell lines used in this study are commonly misidentified lines per ICLAC register.

Animals and other research organisms

Policy information about studies involving animals; ARRIVE guidelines recommended for reporting animal research, and Sex and Gender in Research

Laboratory animals

This study included the following strains of mice: BALB/c (Jackson labs; Strain #000651), NSG (Jackson labs; Strain #005557), and C57BL/6 (Jackson labs; Strain #000664). Mice were generally used for experiments at age 6-12 weeks. Studies were performed with age and sex-matched cohorts. PCNSL model was performed using 8-week old NSG mice (Charles River Labs, Strain: #614).

Mice were maintained at Whitehead Institute under a 12:12 h light/dark cycle (~325 lux during the light phase and <1 lux red light during the dark phase) at an ambient temperature of 21 ± 1 °C and 30–70% relative humidity, with food and water available ad

	libitum, in accordance with all relevant regulations and guidelines in a fully AAALAC-accredited facility. For studies conducted at the Vall d'Hebron Institute of Oncology (VHIO), housing conditions complied with institutional guidelines approved by the CEEA and adhered to Spanish and European Union regulations (Directive 2010/63/EU), which specify temperatures of 20–24 °C and relative humidity of approximately 55 ± 10% under a 12:12 h light/dark cycle.
Wild animals	Not applicable. This study did not involve wild animals.
Reporting on sex	Mice were generally used for experiments at age 6-12 weeks. Studies were performed with age and sex-matched cohorts. In vivo studies using NSG mice were performed with female animals.
Field-collected samples	Not applicable. This study did not involved samples collected from the field.
Ethics oversight	The animal studies reported in this manuscript were approved by the Massachusetts Institute of Technology Committee on Animal Care protocol 2304000511 or the VHIR-CEEA (Comitè d'Ètica d'Experimentació Animal) protocol 27/19.

Note that full information on the approval of the study protocol must also be provided in the manuscript.

Plants

Seed stocks	Not applicable. No plant material was used in this study.
Novel plant genotypes	Not applicable. No plant material was used in this study.
Authentication	Not applicable. No plant material was used in this study.

Flow Cytometry

Plots

Confirm that:

- [x] The axis labels state the marker and fluorochrome used (e.g. CD4-FITC).
- [x] The axis scales are clearly visible. Include numbers along axes only for bottom left plot of group (a 'group' is an analysis of identical markers).
- [x] All plots are contour plots with outliers or pseudocolor plots.
- [x] A numerical value for number of cells or percentage (with statistics) is provided.

Methodology

Sample preparation	Sample preparation is described in detail in Methods section.
Instrument	BD LSR Fortessa (serial number: H17700022), Beckman Coulter CytoFLEX LX (serial number: 37266946)
Software	FlowJo (version 10.9)
Cell population abundance	Not applicable.
Gating strategy	Live cells were first gated based on FSC vs. SSC to exclude debris and dead cells by size and granularity. Subsequently, singlets were gated using FSC-A vs. FSC-H, when applicable, to exclude doublets and aggregates. In some cases a viability dye was used to exclude dead cells (e.g., DAPI). Binding was quantified by mean fluorescence intensity (MFI) of the whole live-cell population. For phagocytosis experiments, macrophages were identified using a fluorophore-conjugated antibody and target cells using CFSE. Phagocytosis was quantified as the percentage of CFSE-positive macrophages within the total macrophage population, and MFI was determined within the full macrophage gate. For experiments evaluating binding to immune subsets, immune cell populations were first gated after live-cell gating using fluorophore-conjugated antibodies, and MFI was then measured within the gated subsets.

- [x] Tick this box to confirm that a figure exemplifying the gating strategy is provided in the Supplementary Information.
